# Müller glial responses compensate for degenerating photoreceptors in retinitis pigmentosa

**DOI:** 10.1038/s12276-021-00693-w

**Published:** 2021-11-19

**Authors:** Yohei Tomita, Chenxi Qiu, Edward Bull, William Allen, Yumi Kotoda, Saswata Talukdar, Lois E. H. Smith, Zhongjie Fu

**Affiliations:** 1 Department of Ophthalmology, Boston Children’s Hospital, Harvard Medical School, Boston, USA; 2 Department of Medicine, Beth Israel Deaconess Medical Center, Harvard Medical School, Boston, USA; 3 Department of Genetics, Harvard Medical School, Boston, USA; 4 Cardiometabolic Diseases, Merck Research Laboratories, Boston, USA; 5 The Manton Center for Orphan Disease, Boston Children’s Hospital, Boston, USA

**Keywords:** Experimental models of disease, Retina

## Abstract

Photoreceptor degeneration caused by genetic defects leads to retinitis pigmentosa, a rare disease typically diagnosed in adolescents and young adults. In most cases, rod loss occurs first, followed by cone loss as well as altered function in cells connected to photoreceptors directly or indirectly. There remains a gap in our understanding of retinal cellular responses to photoreceptor abnormalities. Here, we utilized single-cell transcriptomics to investigate cellular responses in each major retinal cell type in retinitis pigmentosa model (P23H) mice vs. wild-type littermate mice. We found a significant decrease in the expression of genes associated with phototransduction, the inner/outer segment, photoreceptor cell cilium, and photoreceptor development in both rod and cone clusters, in line with the structural changes seen with immunohistochemistry. Accompanying this loss was a significant decrease in the expression of genes involved in metabolic pathways and energy production in both rods and cones. We found that in the Müller glia/astrocyte cluster, there was a significant increase in gene expression in pathways involving photoreceptor maintenance, while concomitant decreases were observed in rods and cones. Additionally, the expression of genes involved in mitochondrial localization and transport was increased in the Müller glia/astrocyte cluster. The Müller glial compensatory increase in the expression of genes downregulated in photoreceptors suggests that Müller glia adapt their transcriptome to support photoreceptors and could be thought of as general therapeutic targets to protect against retinal degeneration.

## Retinitis pigmentosa: Focus on genes suggests possible treatments

Insights into the genetic and metabolic changes in retinitis pigmentosa, a rare genetic disorder that leads to increasingly worse vision, suggest a new approach for treating degenerative diseases of the retina. Researchers in the US led by Zhongiie Fu at Boston Children’s Hospital/Harvard Medical School, Boston, analyzed the activity of genes in the retinal cells of mice used to model retinitis pigmentosa in humans. They detected a significant decrease in the activity of several genes involved in light reception, subsequent reduced nerve signalling activities and metabolism in the rod and cone cells of the retina. These cells facilitate vision at low and high light levels, respectively. Increased gene activity in retinal cells called the Müller glial cluster partially compensated for these defects. The results suggest possible gene and protein targets for new treatments.

## Introduction

Retinitis pigmentosa (RP) is a rare retinal disorder with photoreceptor (rod and cone) loss, and most gene mutations (>150) associated with RP are in rods^1,2^. It is difficult to develop gene therapies and run trials for each different genetic cause of RP due to the multitude of mutations, some of which are still unidentified. If a common pathway contributing to photoreceptor degeneration could be targeted, retinal function might be maintained in all types of RP. Evidence suggests that glucose shortage in photoreceptors leads to their demise, and stimulating the mammalian target of rapamycin complex 1 (mTORC1) pathway delays cone degeneration^3–5^. Elucidating the common cellular and molecular pathways supporting photoreceptor function would be useful for the development of a generalized therapeutic approach for treating multiple retinal degenerative diseases.

Here, we analyzed the gene profile of each major retinal cell type (rods, cones, bipolar cells, amacrine cells, and retinal glia (Müller glia/astrocytes)) using single-cell RNA-seq (scRNA-seq) in a RP model (P23H) mice. These mutant mice, which have an amino acid substitution of proline to histidine in codon 23 of rhodopsin (the most common mutation responsible for autosomal-dominant RP in the USA)^6^, show decreased rod responses followed by reduced cone responses, similar to human patients^7^.

## Materials and methods

### Study approval

All animal studies adhered to the Association for Research in Vision and Ophthalmology Statement for the Use of Animals in Ophthalmic and Vision Research and were approved by the Institutional Animal Care and Use Committee at Boston Children’s Hospital.

### Optical coherence tomography (OCT)

P23H homozygous mice (stock 017628, Jackson Laboratory, USA) were bred with C57BL/6J (wild-type, WT, stock 000664, Jackson Laboratory) mice to generate heterozygous (het) P23H mice. Het P23H mice were bred with C57BL/6J mice to generate littermate P23H (refer to as het P23H) and WT mice. Mouse genotypes were assessed by Transnetyx, Inc. (USA). Spectral-domain OCT under the guidance of bright-field live fundus imaging^8^ was performed (Micron IV, Phoenix Research Laboratories, USA) in mice at postnatal week (PW) 4, when OCT was first feasible. The thickness of the inner plexiform layer (IPL), inner nuclear layer (INL), outer nuclear layer (ONL), and photoreceptor inner/outer segment (IS/OS) was measured using ImageJ. The thickness was measured in the middle retina between the central optic nerve head and the edge of the image on both the nasal and temporal sides, and the thickness of retinal layers was then averaged for each eye.

### Immunohistochemistry

At PW2, PW4, and PW7, mouse eyeballs were cross-sectioned at 14 µm using a Cryostat (Leica CM3050 S). Retinal cross-sections including the optic nerve head were used for immunohistochemistry^8^. The sections were stained with primary antibodies against rhodopsin (1:500, MABN15, Millipore, USA), cone arrestin (1:500, AB15282, Millipore), GFAP (1:500, ab4674, Abcam, USA), calretinin (1:1000, AB1550, Millipore), and PKCα (1:500, sc-208, Santa Cruz, USA) overnight at 4 °C. The sections were then counterstained with the corresponding fluorescent secondary antibody and covered in mounting medium with 4′,6-diamidine-2′-phenylindole dihydrochloride (DAPI for cell nuclei, H-1200, Vector Laboratories, USA). The immunostaining signals were visualized with a Zeiss confocal microscope at 200X magnification.

### Single-cell transcriptomics

Retinas from P23H and littermate WT mice at PW7 were dissected to remove the anterior segment and retinal pigment epithelium (RPE) cells. Single-cell suspensions were prepared from the mouse retinas using a Worthington papain dissociation system (PDS, Worthington, USA). A retinal cell barcoded inDrop library was prepared at the Single Cell Core at Harvard Medical School (HMS)^9,10^ and sequenced at the Center for Cancer Computational Biology, Dana Farber Cancer Institute, on a NextSeq 500 with paired-end 75 bp reads. The raw data were deposited at the SRA and can be accessed with NIH BioProject number PRJNA707351.

#### Single-cell suspension preparation

A Worthington PDS was prepared. The papain powder was reconstituted in 5 ml of EBSS solution, the ovomucoid powder was reconstituted in 32 ml of EBSS solution, and the DNase I powder was reconstituted in 500 µl of EBSS solution. In a 15 ml Corning tube, 2.5 ml of reconstituted papain solution was mixed gently with 250 µl of reconstituted DNase I solution and 7.5 ml of EBSS. One milliliter of papain mixture was placed into a separate 15 ml tube. In a 15 ml Corning tube, 0.3 ml of reconstituted ovomucoid solution was mixed gently with 150 µl of DNase I and 2.7 ml of EBSS solution. The solution was buffered with 95% oxygen/5% CO_2_ until the pH reached 7.2, sealed with paraffin and maintained at 37 °C in a water bath before use.

Two retinas isolated from the same mouse were pooled together and minced with a blade into fine pieces. The retinas were then transferred into 1 ml of papain mixture, pipetted 3–4 times to break the retina into small pieces, and immediately transferred to 9 ml of papain solution. The cap was sealed with paraffin, and the tube was incubated in a 37 °C water bath for 45 min. The mixture was triturated with a 10 ml pipette to resuspend the pellet and centrifuged at 300*g* for 5 min at room temperature. The supernatant was removed, and the pellet was resuspended in the ovomucoid mixture and centrifuged at 300*g* for 5 min at room temperature. The supernatant was removed, and the pellet was suspended in 1 ml of ice-cold 0.2 µm-filtered 1% BSA in 1X PBS. The single-cell suspension was filtered through a 40 µm cell strainer, and cell viability was examined using Trypan blue. We noticed that cell viability depended on the age of the mice and the degenerative status of the retinas. We normally observed > 90% viability for neonatal retinas and less (~80%) viability for adult and degenerating retinas.

#### Single-cell library preparation

For inDrop scRNA-seq, the cells were encapsulated in droplets, and libraries were made as previously described^9,10^ with the following modifications to the primer sequences:

RT primers on hydrogel beads: 5′-CGATTGATCAACGTAATACGACTCACTATAGGGTGTCGGGTGCAG[bc1,8nt]GTCTCGTGGGCTCGGAGATGTGTATAAGAGACAG[bc2,8nt]NNNNNNTTTTTTTTTTTTTTTTTTTV-3′;R1-N6 primer sequence (step 151 in the library prep protocol in^9^): 5′-TCGTCGGCAGCGTCAGATGTGTATAAGAGACAGNNNNNN-3′;

PCR primer sequences (steps 157 and 160 in the library prep protocol in^9^): 5′- AATGATACGGCGACCACCGAGATCTACACXXXXXXXXTCGTCGGCAGCGTC-3′ (where XXXXXX is an index sequence for multiplexing libraries) and

5′-CAAGCAGAAGACGGCATACGAGATGGGTGTCGGGTGCAG-3′.

#### Sequencing

The sequencing library samples were submitted to the Center for Cancer Computational Biology for quality control and sequencing. The library quality control included size validation using a Bioanalyzer (Agilent Technologies, USA) and verification of the ligated libraries using qPCR (ABI 7900 HT machine using Kapa kits). The libraries were deemed suitable for sequencing and pooled together at equal concentrations to form a sequencing pool at 2 nM. The libraries had unique indices attached. The sequencing library pool was denatured and diluted to 2 pM before being loaded onto the sequencer. Sequencing was performed on a NextSeq 500 (Illumina, USA) in paired-end mode with 61 cycles for Read 1 and 14 cycles for Read 2 and dual indexing of 8 cycles for each index read. The data were obtained in FASTQ format.

#### Analysis

The scRNA-seq data were analyzed as previously described^11,12^ with modifications. Briefly, read preprocessing and alignment to the mouse transcriptome (GRCm38.81) were performed following an inDrop bioinformatics pipeline^10^. Cells with insufficiently expressed genes (<500 genes) or a large fraction of mitochondrial genes (6 median absolute deviations larger than the median) were not considered for further analyses. Potential doublet cells (two cells within one droplet) arising from single-cell isolation were computationally removed following a pipeline to estimate the doublet score^13^. Cells with a doublet score of > 99% were considered putative doublets and left out of downstream analyses. The number of cells from each mouse retinal sample included in the analysis (Supplementary Table 1) and the total number of cells with > 500 detectable genes in each cluster (Supplementary Table 2) are shown. We did not anticipate and did not observe a significant batch effect in principal component analysis. Normalization and dimension reduction using Seurat 3 were performed^14^. Briefly, we normalized, log-transformed, and scaled the expression of each cell using the default settings of the NormalizeData, FindVariableFeatures and ScaleData functions (Seurat 3). Principal component analysis was subsequently computed with the number of principal components arbitrarily set to 100. The number of clusters was determined, and tSNE clustering was performed with the top 30 principal components using the default settings of the FindNeighbors, FindClusters, and RunTSNE functions. The cell type was identified by first comparing the scaled expression profile to the mouse cell atlas^15^ and then confirming the expression of the known cell type-specific marker genes. Clusters in which the vast majority of cells were the same cell type were subjected to downstream analyses, except for Cluster 12 for biological reasons discussed in the “Results”. Clusters consisting of the same cell type were combined for further analyses.

Differential gene expression analyses were performed as previously reported^12^ using the glmFit model from the edgeR package^12,16–19^. We estimated the negative binomial distribution dispersion before modeling using the estimateDisp and glmFit functions and computed the log2(fold change), *P*, and false discovery rate-adjusted *P* values as described^12^. A *P* value < 0.001 was considered a cutoff to define differentially expressed genes. Differentially expressed genes were subjected to Gene Ontology (GO) analysis using clusterProfiler^20^ and gene-set enrichment analysis^21,22^.

### Real-time PCR

Total retinal RNA from PW7 P23H and WT mice was extracted^23,24^. RNA was extracted using a PureLink^®^ RNA Mini Kit (#12183018 A, Ambion, USA), and cDNA was synthesized using an iScript^TM^ cDNA synthesis kit (#1708891, Bio-Rad, USA). qPCR was performed using the following primers: *rhodopsin (Rho)*: 5′-TCATGGTCTTCGGAGGATTCAC-3′, 5′-TCACCTCCAAGTGTGGCAAAG-3′; phosphodiesterase 6B (*Pde6b)*: 5′-CCCCTGACTCTGAGATCGTC-3′, 5′-TGATCACAGCCACGACATCT-3′; and phosphodiesterase 6 G *(Pde6g)*: 5′-CAGCCTGACAGAGTCCAGAA-3′, 5′-GCTTGCTCTTGAACTGCCTT-3′. Gene expression was analyzed using an Applied Biosystems 7300 Sequence Detection System with a SYBR Green Master Mix Kit. The number of amplified cDNA copies was normalized to that of the housekeeping gene *cyclophilin A (CycloA)* (5′-CAGACGCCACTGTCGCTTT-3′, 5′-TGTCTTTGGAACTTTGTCTGCA-3′) using the ΔΔCt method. The relative mRNA levels are presented as the fold change versus WT mice.

### Western blot analysis

The levels of rhodopsin (RHO) were assessed in protein lysates from PW7 P23H and WT mouse retinas using a RHO antibody (1:1000, MABN15, Millipore, USA) in 5% nonfat milk (#M0841, Labscientific, USA) overnight at 4 °C^8,12^. The signals were detected using 1:2500 dilutions of corresponding horseradish peroxidase-conjugated secondary antibodies and enhanced chemiluminescence (ECL) reagent (#32106, Thermo Scientific, USA), and then the digital images were visualized with an Azure Biosystems instrument. β-ACTIN (1:5000, A1978, Sigma, USA) was used as an internal control.

### Statistics

All data are presented as mean ± SEM. A two-tailed unpaired *t* test was used for comparison of the results (Prism v9.0; GraphPad Software, Inc., San Diego, CA, USA). The threshold for statistical significance (*α*) was set at 0.05.

## Results

### Time course of photoreceptor degeneration and retinal gliosis in P23H mice

**Fig. 1:**
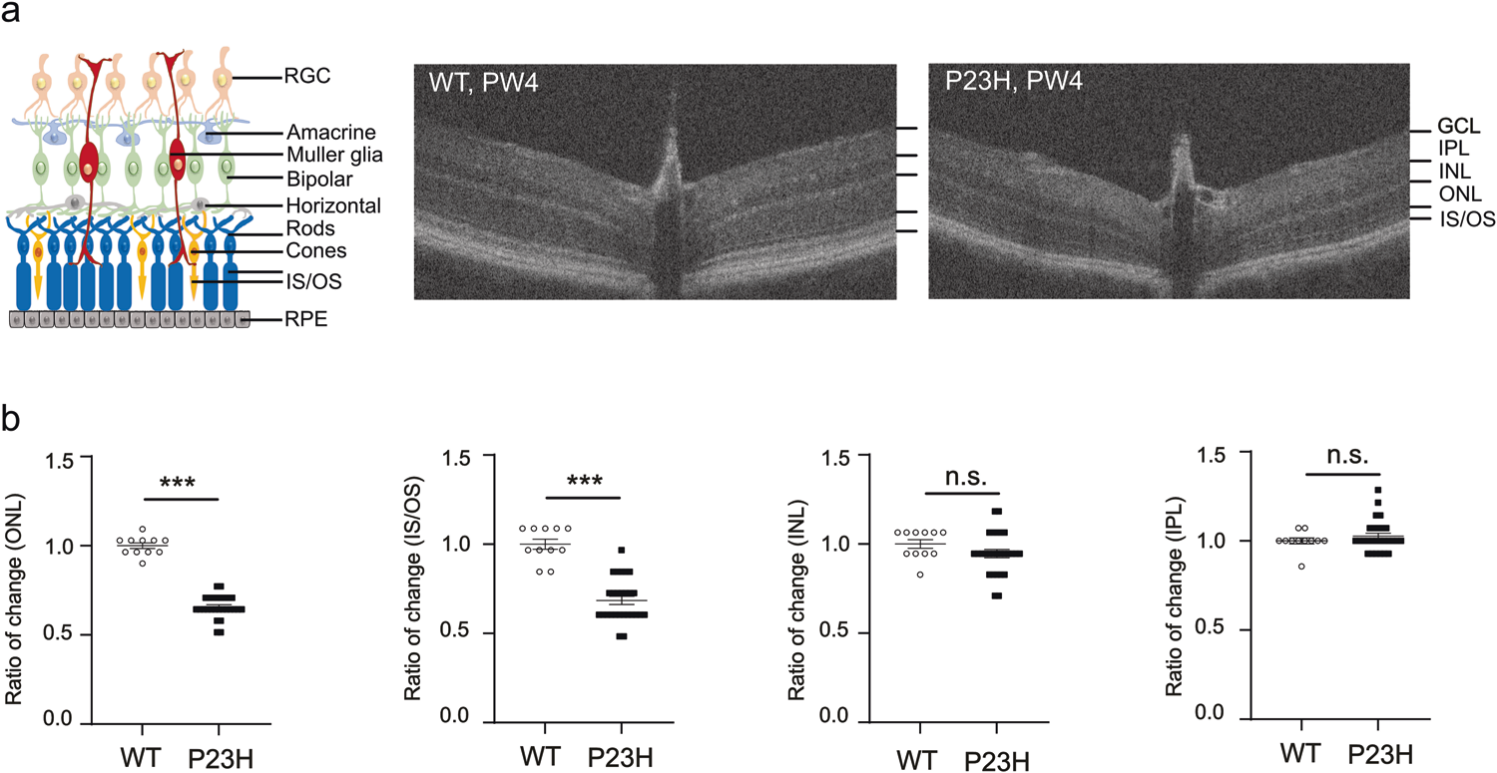
Decreased photoreceptor layer thickness in P23H mice at PW4. **a** Schematic of retinal structure and representative OCT images from the nasal to temporal sides of WT and P23H mouse retinas at PW4. GCL, ganglion cell layer; IPL, inner plexiform layer; INL, inner nuclear layer (amacrine, bipolar, horizontal, and Müller glial cell nuclear layer); ONL, outer nuclear layer (photoreceptor nuclear layer); IS/OS, photoreceptor inner/outer segment; RPE, retinal pigment epithelium. The edge of each layer on the OCT images is labeled with a black line. **b** Decreased thickness of the ONL and IS/OS in P23H vs. WT retinas. The fold change in thickness compared to that of the WT mice was calculated. *n* = 11 eyes (WT), *n* = 27 eyes (P23H). The data are presented as mean ± SEM. Unpaired *t* test. ^***^P < 0.001, n.s., not significant.

OCT was first used to measure retinal thickness in P23H and WT mice at PW4 in vivo (Fig. 1a). We found an ~35% decrease in ONL (photoreceptor nuclear layer) thickness and an ~30% decrease in the photoreceptor IS/OS layer in P23H vs. WT mice (Fig. 1b), suggesting that photoreceptor degeneration occurs earlier than PW4. There was no significant change in the thickness of the INL (inner neuronal nuclear layer) or IPL (layer composed of synapses connecting inner neurons with retinal ganglion cells (RGCs); Fig. 1b).

**Fig. 2:**
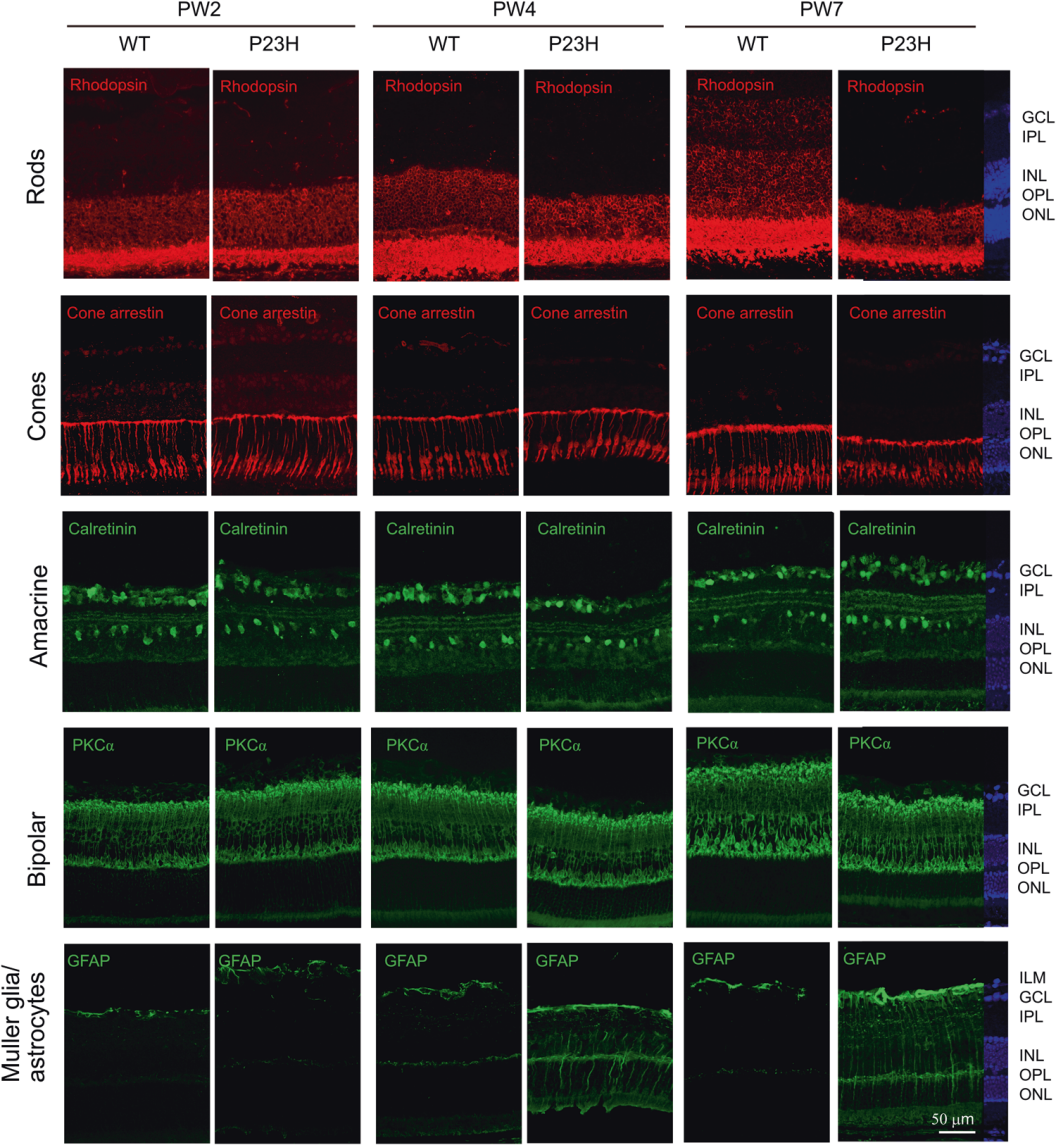
Retinal neuronal degeneration and Müller glia/astrocyte gliosis occurred in P23H mice. Rod degeneration (rhodopsin) was observed at PW4 along with decreased thickness of the ONL and IS/OS, which was further reduced at PW7. Rhodopsin was also observed in the INL at PW7 in WT retinas but not in P23H retinas. Cone degeneration (cone arrestin) was also observed at PW4 with shortened outer segments, with further disruption at PW7. Retinal gliosis (GFAP) was observed at PW4 and to a greater extent at PW7. No significant changes were observed for amacrine cells (calretinin) or rod bipolar cells (PKCα). DAPI staining was used to show cell nuclei. ILM, inner limiting membrane; GCL, ganglion cell layer; IPL, inner plexiform layer; INL, inner nuclear layer; OPL, outer plexiform layer; ONL, outer nuclear layer. Images were taken at 200X magnification. Scale bar, 50 μm.

To better understand the time course of morphological changes in P23H retinas, immunohistochemistry for major retinal cell types was conducted in P23H and littermate WT mouse retinal cross-sections at PW2, PW4, and PW7 (Fig. 2). Immunoreactivity for the rod marker rhodopsin^8^ was mainly observed in the ONL and IS/OS region in WT retinas at PW2, PW4, and PW7. P23H mice showed decreased rhodopsin immunostaining in the ONL and IS/OS junction at PW4, with further decreases at PW7. The cone marker cone arrestin^8^ was localized in the cone cell nucleus at the outer margin of the ONL, in cell processes across the INL extending to the OPL, and in the IS/OS region in WT retinas. P23H retinas had a structure comparable to that of WT at PW2. The cone IS/OS in P23H retinas was shorter than that in WT retinas at PW4 and was even more disrupted at PW7. P23H retinas had calretinin (amacrine marker^25,26^) and PKCα (rod bipolar cell marker^26,27^) immunoreactivity comparable to that of WT retinas. GFAP immunoreactivity was observed in astrocytes along the ILM and faintly present in the OPL in WT retinas. In P23H retinas, GFAP immunoreactivity was observed in some cell processes across the entire retina at PW4 and was stronger at PW7.

### Disrupted photoreceptor gene profile in P23H mice at PW7

**Fig. 3:**
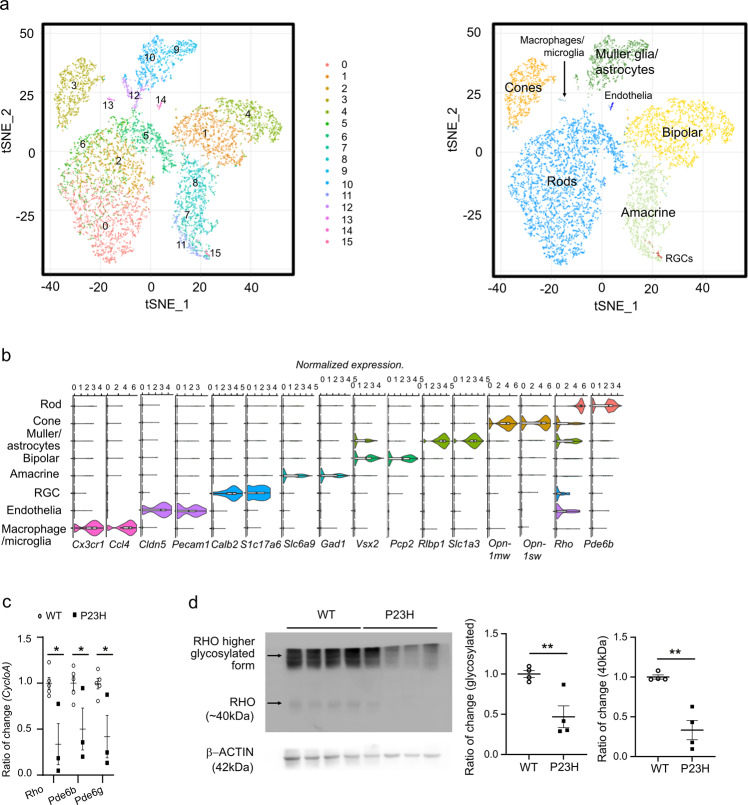
Single-cell transcriptomics of retinal cells from P23H vs. WT mice at PW7. **a** tSNE projection of different color-coded retinal cell types from P23H vs. WT littermate controls. *n* = 4 mice per group, littermates. **b** Violin plots of normalized marker gene expression for different cell types, as follows: *Rho* and *Pde6b* (rods), *Opn1mw* and *Opn1sw* (cones), *Rlbp1* and *Slc3a1* (Müller glia and astrocytes), *Vsx2* and *Pcp2* (bipolar cells), *Slc6a9* and *Gad1* (amacrine cells), *Calb2* and *Slc17a6* (retinal ganglion cells, RGCs), *Cldn5* and *Pecam1* (endothelial cells), and *Cx3cr1* and *Ccl4* (macrophages and microglia). **c** Validation of *Rho, Pde6b*, and *Pde6g* expression in P23H vs. WT mouse retinas at PW7 with qPCR. *n* = 5 mice for WT, *n* = 3 mice for P23H. The data are presented as mean ± SEM. Unpaired *t* test, ^*^P < 0.05. **d** Validation of RHO levels in P23H vs. WT mouse retinas at PW7 with western blot analysis. *n* = 4 mice per group. The data are presented as mean ± SEM. Unpaired *t* test, ^**^P < 0.01.

To understand the underlying cellular responses in different retinal cell types during photoreceptor degeneration, we conducted single-cell transcriptomics on P23H and WT retinas at PW7, when strong rod and cone structural disruption was observed. First, we found 15 tSNE cell clusters with distinct expression profiles (Fig. 3a). Cell types were identified by correlating the expression profiles of individual cells with the profile from the Mouse Cell Atlas, independently validated by the expression of known markers and collapsed into 8 cell types (Fig. 3b). Cluster 12 had a mixed expression of rod, cone, and Müller glial marker genes and was excluded from separate analyses (below), while other clusters were combined into known cell types. The expression of *Rho* (log*FC* = −0.82387, *P* < 0.001), *Pde6b* (log*FC* = −0.49883, *P* < 0.001), and *Pde6g* (log*FC* = −0.52962, *P* < 0.001) was decreased in the rod cluster, in line with the decreased total mRNA levels of *Rho, Pde6b*, and *Pde6g* in P23H vs. WT retinas at PW7 in the qPCR validation (Fig. 3c) and the decreased RHO levels in P23H vs. WT retinas at PW7 in the western blot validation (Fig. 3d). These findings suggested that scRNA-seq analysis reliably recapitulated the known and expected underlying differences between P23H and WT retinas.

**Fig. 4:**
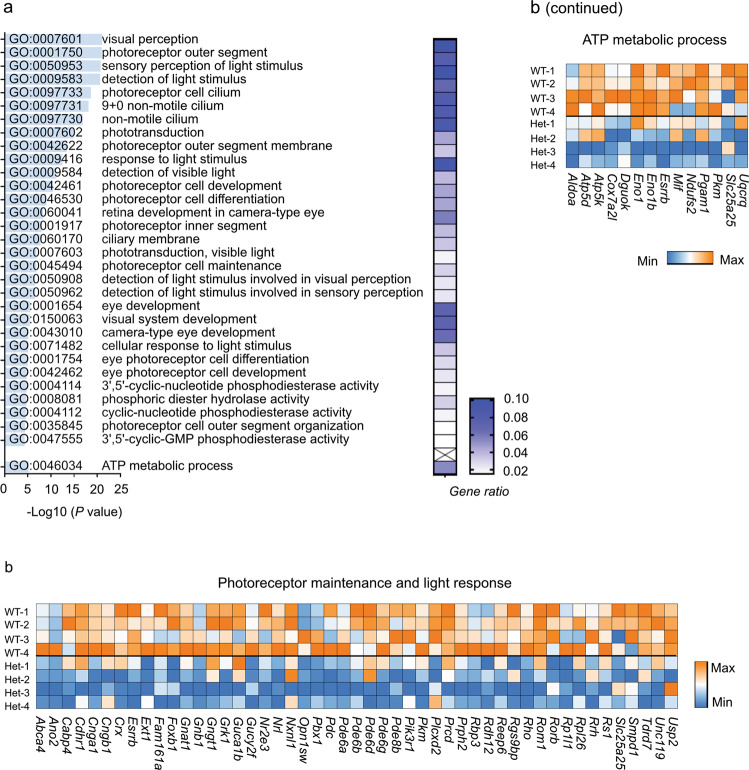
Downregulated pathways in rods in P23H mice at PW7. **a** The most downregulated genes in the rod cluster of P23H retinas were associated with photoreceptor maintenance, and light-response Gene Ontology terms (phototransduction, IS/OS segment, photoreceptor cell cilium, and photoreceptor development) and ATP metabolic process-related Gene Ontology terms. The *P* values for the enriched Gene Ontology (GO) terms are shown in bar graphs (*P* < 0.001). The gene ratio for each pathway is shown in the heatmap. **b** Heatmap of genes involved in photoreceptor maintenance, light responses, and ATP metabolic processes in the rod population of P23H mouse retinas.

**Fig. 5:**
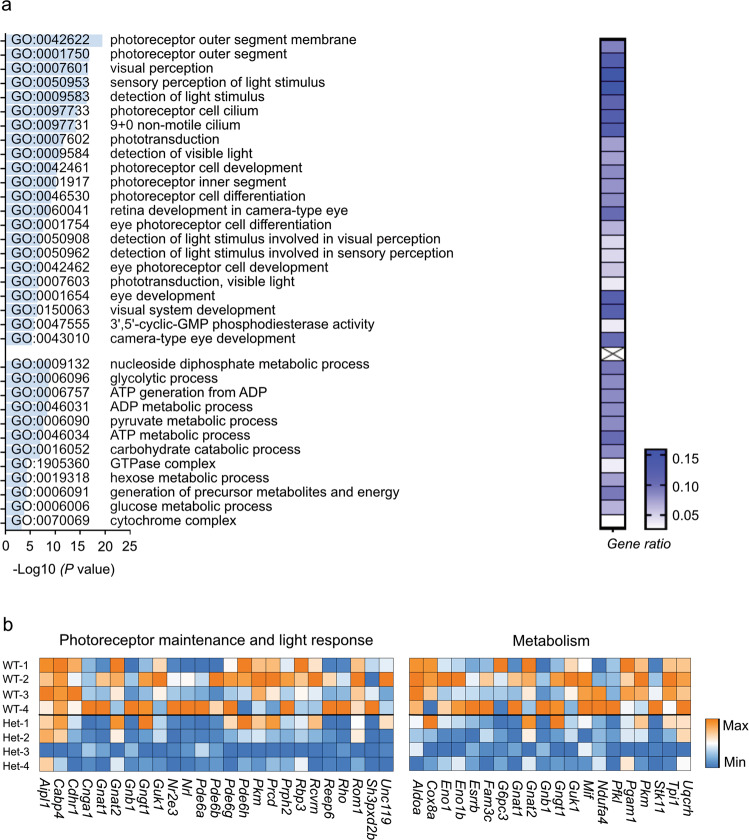
Downregulated pathways in cones in P23H mice at PW7. **a** The most downregulated genes in the cone population of P23H retinas were associated with photoreceptor maintenance- and light response-related Gene Ontology terms (phototransduction, IS/OS segment, photoreceptor cell cilium, and photoreceptor development) and metabolism-related Gene Ontology terms. The *P* values for the enriched Gene Ontology (GO) terms are shown in bar graphs (*P* < 0.001). The gene ratio for each pathway is shown in the heatmap. **b** Heatmap of genes involved in photoreceptor maintenance, light responses, and metabolism in the cone population of P23H mouse retinas.

To further investigate the cell type-specific alterations in P23H retinas, we performed GO analyses (Supplementary Tables 3–8) and found that in rods, there was a significant decrease in the expression of genes associated with phototransduction, the IS/OS segment, photoreceptor cell cilium, and photoreceptor development (Fig. 4a, b), in line with the loss of the rod IS/OS during degeneration (Fig. 2). There was also a decrease in ATP metabolic process gene expression (Fig. 4a, b). Similarly, in cones, we observed a large decrease in the expression of genes associated with phototransduction, the IS/OS segment, photoreceptor cell cilium, and photoreceptor development (Fig. 5a, b). There was also a large decrease in the expression of genes involved in metabolic pathways, including glycolysis and ATP generation (Fig. 5a, b). For example, the glycolytic enzyme pyruvate kinase muscle (*Pkm*) was decreased in the rod (log*FC* = −0.40064, *P* < 0.001) and cone clusters (log*FC* = −0.68706, *P* < 0.001). Genetic deficiency of photoreceptor-specific *Pkm2* leads to photoreceptor degeneration in mice^28,29^. In amacrine and rod bipolar cells, the changes in gene expression were relatively mild (Supplementary Tables 7 and 8), in line with the lack of morphological changes (Fig. 2). These findings suggest that degenerating rods and cones experience compromised photoreceptor function and a shortage of metabolic needs.

### Retinal glial compensatory responses in P23H mice at PW7

**Fig. 6:**
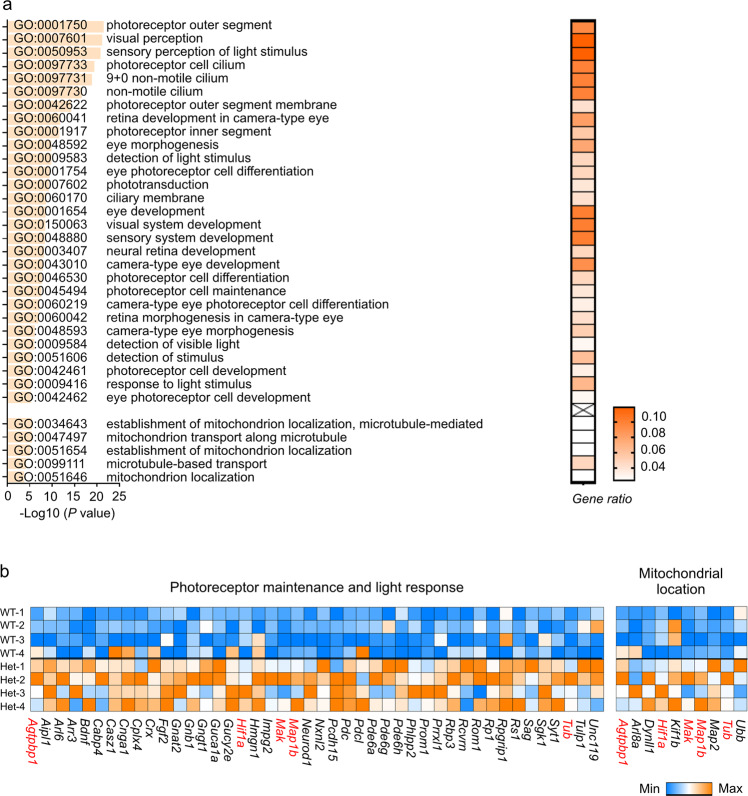
Upregulated pathways of Müller glia/astrocytes in P23H mice at PW7. **a** The most upregulated genes in the Müller glia/astrocyte population of P23H retinas were associated with photoreceptor maintenance-related Gene Ontology terms (phototransduction, IS/OS segment, photoreceptor cell cilium, and photoreceptor development) and mitochondrial location-related Gene Ontology terms. The *P* values for the enriched Gene Ontology (GO) terms are shown in bar graphs (*P* < 0.001). The gene ratio for each pathway is shown in the heatmap. **b** Heatmap of genes involved in photoreceptor maintenance, light responses, and mitochondrial location in the Müller glia/astrocyte population of P23H mouse retinas. Repeated genes observed in both categories are labeled in red.

Remarkably, we observed a compensatory gene expression profile pattern in retinal glia (Müller glia/astrocytes). The expression of genes associated with phototransduction, the IS/OS segment, photoreceptor cell cilium, and photoreceptor development in retinal glia was increased in P23H (Fig. 6a). It is worth noting that the expression of genes involved in mitochondrial localization and transport was also increased (Fig. 6b). For example, the expression of kinesin family member 1B (*Kif1b*), which works as a monomeric motor for anterograde transport of mitochondria^30^, was increased (log*FC* = 0.469942, *P* < 0.001); that of microtubule-associated protein 2 (*Map2*), which binds to mitochondria and facilitates the interaction of mitochondria with microtubules^31,32^, was also increased (log*FC* = 0.546072, *P* < 0.001). Despite the nature of these specific genes, there appeared to be a collective compensatory transcriptomic response from retinal glia to the loss of photoreceptor function and metabolic needs in rods and cones, a cell type-specific effect that could have been masked in bulk RNA-seq experiments.

**Fig. 7:**
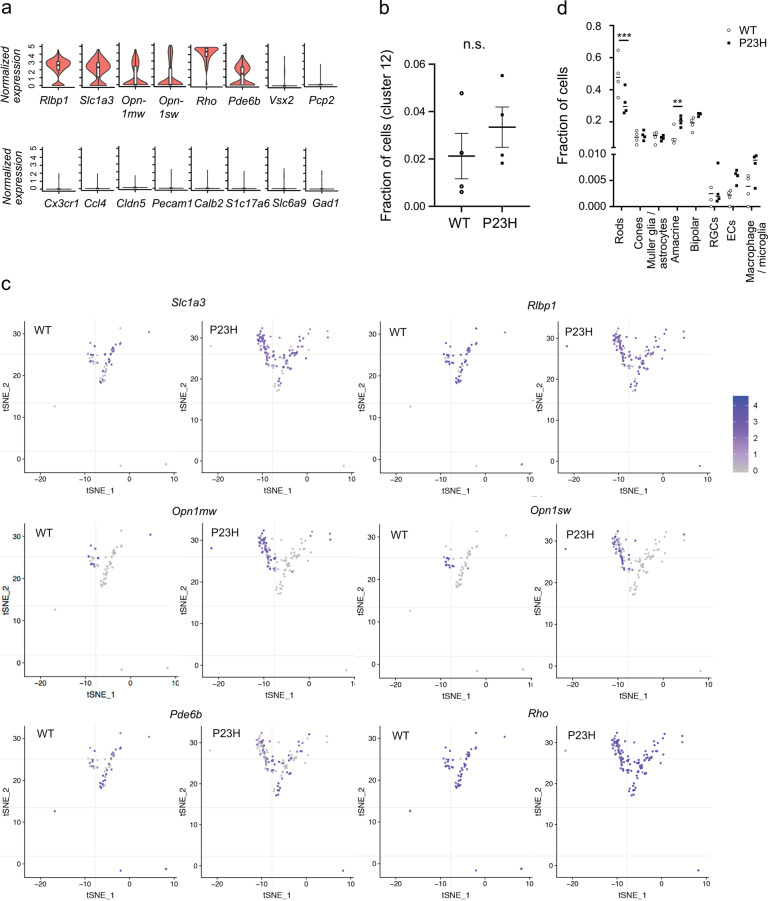
Analysis of the gene profile in Cluster 12 in P23H vs. WT mice. **a** Violin plots of normalized marker gene expression in Cluster 12. Cluster 12 was positive for *Rho* and *Pde6b* (rods), *Opn1mw* and *Opn1sw* (cones), and *Rlbp1* and *Slc3a1* (Müller glia and astrocytes). **b** Fraction of Cluster 12 cells in P23H het vs. WT littermate mice. *n* = 4 mice per group. The data are presented as mean ± SEM. n.s., not significant, two-tailed unpaired *t* test. **c** Heatscatter plot of normalized expression for the indicated genes in individual cells in Cluster 12. The cells are presented in the tSNE projection and color-coded by the normalized expression from gray (low) to blue (high). **d** Comparison of the fraction of cells in major retinal clusters in P23H vs. WT mice (with Cluster 12 excluded). ^***^P < 0.001, ^**^P < 0.01, unpaired *t* test.

We then looked into Cluster 12, which was positive for *Rho* and *Pde6b* (rod markers), *Opn1mw* and *Opn1sw* (cone markers), and *Rlbp1* and *Slc3a1* (Müller glia/astrocytes) and appeared to be a mixed-cell-type cluster (Fig. 7a). We first confirmed that Müller glia/astrocyte markers were coexpressed with rod and cone markers at the single-cell level, thus excluding the possibility that this cluster was a mixed population of Müller glia/astrocytes, cones, and rods. We found that P23H vs. WT retinas had a trend of an increased fraction of cells in Cluster 12 (Fig. 7b). We noted that there were two populations of cells in Cluster 12 (Fig. 7c). One was positive for Müller glia/astrocyte and rod markers, and the other was positive for Müller glia/astrocyte, rod, and cone markers. Of note, we could not fully exclude the possibility that this cluster consisted of doublet cells. However, this was unlikely, as we carefully assessed the doublet cell score, which assesses the possibility of doublet cells by features such as total RNA count, and filtered out the potential doublet cells in the preprocessing step as a quality control measure. Interestingly, we found that P23H retinas showed a trend of an increase in the subpopulation of Müller glia/astrocytes that expressed rod and cone markers (Fig. 7c). In addition, to rule out the possibility that Cluster 12 was a mixed population of Müller glia, rods, and cones, we confirmed that Müller glial markers were coexpressed with rod or cone markers in the same set of Cluster 12 cells in a set of two-dimensional correlation *xy* plots (Supplementary Fig. 1). The results were consistent with the proposed idea that retinal glia might produce photoreceptor proteins under normal conditions and that their production might increase as a compensatory response to photoreceptor degeneration.

We also observed a decreased fraction of cells in rods and an increased fraction of cells in amacrine cells in P23H vs. WT retinas (Fig. 7d). These findings correlated with the loss of rods and cones in P23H mice with more aggressive rod degeneration. The increase in amacrine cells might have been a result of decreased total retinal cell numbers in P23H mice. The comparable fractions of cells in retinal glia might have been due to more retinal glial cells expressing rod and cone markers in Cluster 12, which was not included in the Müller glia/astrocyte cluster.

### Transcription factors identified with gene-set enrichment analysis

**Fig. 8:**
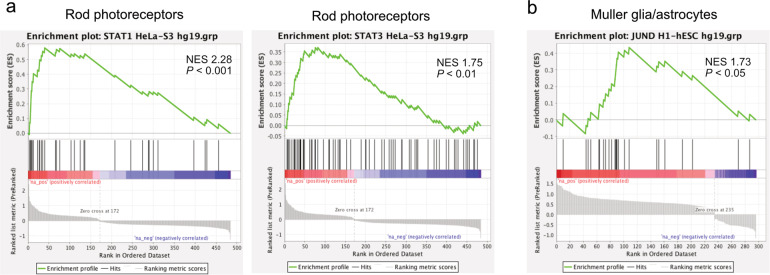
Gene-set enrichment analysis of retinal transcription factors in P23H vs. WT mice. Gene-set enrichment analysis was performed using Encode ChIP seq gene sets. Gene-set enrichment analysis revealed significant enrichment of STAT1 and STAT3 binding motifs in rods (**a**) and JUND in Müller glia/astrocytes (**b**) in the differentially expressed genes induced in P23H mice (compared to littermate WT controls).

To predict the transcription factor(s) modulating photoreceptor and retinal glial responses in P23 mice, we conducted gene-set enrichment analysis to examine the potential common binding motifs of transcription factors associated with the differentially expressed genes affected in P23H mice (Supplementary Table 9). STAT1 (Fig. 8a, NES = 2.28, *P* < 0.001) and STAT3 (Fig. 8a, NES = 1.75, *P* < 0.01) motifs were enriched in the genes with increased expression in P23H rods. We did not observe enriched motifs associated with decreased gene expression in rods and cones. JUND motifs (Fig. 8b, NES = 1.73, *P* < 0.05) were enriched and associated with increased expression of genes in P23H Müller glia/astrocytes.

## Discussion

We found that photoreceptors had decreased expression of genes associated with metabolic pathways and might therefore experience a shortage in energy supply during degeneration. Concomitantly, Müller glia responded to photoreceptor degeneration with increased expression of genes involved in photoreceptor maintenance and mitochondrial localization and transport. These findings suggest that we may need to revise our current understanding of the role of Müller glia in the response to photoreceptor loss.

Evidence suggests that targeting photoreceptor metabolism may help protect the neurovasculature in diseases of the mouse retina^23,33^. Cone loss is associated with cone starvation (and oxidative stress) in mouse RP with mutations in rod-specific genes^3^. Suppression of endogenous insulin increases cone loss, and exogenous insulin treatment increases cone survival^3^. Rod-derived cone viability factor increases glucose entry into cones and maintains cone metabolic function^4^. Loss of rods deprives cones of these survival factors. However, our current knowledge of this field is still very limited. We found that the metabolic genes with decreased expression in P23H mice were similar in rod and cone clusters and were mostly directly involved in glucose metabolism. These findings suggest that rods and cones may share a similar altered metabolic signature during photoreceptor degeneration in mouse RP, potentially allowing therapeutic interventions targeting common pathways to improve energy production. However, continuous activation of mTORC1 in photoreceptors increases cell metabolic needs, mimics glucose shortage, and eventually leads to drusen-like deposits in mouse retinas^5^, suggesting that stimulation of glycolysis in photoreceptors is likely to be tightly regulated and that overstimulation may be deleterious. We also need to expand our current knowledge of the metabolic links between photoreceptors and surrounding cells^34^. RPE cells in vitro preferably utilize lactate as an energy substrate and shunt glucose to photoreceptors^35^. Increasing RPE glucose use causes a glucose supply shortage to photoreceptors and induces photoreceptor death in mice^36^. These reports suggest that with any intervention modulating metabolism, we need to consider the implications of the metabolic needs of (and interactions among) different types of retinal cells.

Interestingly, we found adaptive and compensatory gene expression responses in retinal glia (Müller glia/astrocytes). We speculate that (1) retinal glia may produce and supply these proteins to photoreceptors as a rescue process or (2) retinal glia may respond directly to light stimulation to preserve aspects of retinal function as they begin to produce photoreceptive pigments. Müller glia produce rhodopsin in mouse RP *rd1* retinas^37^ and provide 11-*cis*-retinol for photoreceptors to maintain normal function^38,39^, indicating that the first hypothesis might be valid. This hypothesis is in line with our observations of the cells in Cluster 12, which were positive for gene markers of retinal Müller glia/astrocytes, rods, and cones (Fig. 7, Supplementary Fig. 1). Müller glia also have the potential to re-enter the cell cycle and differentiate into retinal neurons in zebrafish^40^. Müller glia in the mammalian retina have regenerative capacity, although regeneration does not occur spontaneously^41,42^. Hippo signaling induces Müller glia to attain a highly proliferative and progenitor cellular stage in mice^41^. Gene transfer of beta-catenin stimulates Müller glial proliferation and reprogramming to generate rods in mice^42^. Limited neurogenesis of Müller glia is observed in response to retinal injury^43^. Our results suggest that Müller glia may be able to undergo neurogenesis in response to photoreceptor loss in P23H mice. A better understanding of Müller glial activation during photoreceptor degeneration in mammalian retinas is needed.

Müller glia also contribute to the survival of photoreceptors through metabolic support^34^. In healthy retinas, Müller glia have a very homogeneous metabolic signature, while in early retinal degeneration, they are heterogeneous, consistent with the ability of Müller glial cells to adjust metabolically in order to “nurse” healthy or degenerating photoreceptors^44^. We found increased expression of genes involved in mitochondrial localization and microtubule-based transfer in Müller glia/astrocytes. We speculate that (1) Müller glia may transfer mitochondria to degenerating photoreceptors to increase energy production or (2) Müller glia might increase their own energy production, in line with potential neurogenesis. In the brain, astrocytes transfer mitochondria to neurons for neuroprotection after stroke; however, the mechanism behind astrocytic mitochondrial release and entry into neurons is unknown^45^. In the retina, astrocytes degrade damaged mitochondria released from RGCs^46^. It is not known whether Müller glia can transfer mitochondria to metabolically stressed photoreceptors. Further investigation is clearly needed.

In addition to exploring common pathways that may benefit retinal function, we examined potential transcription factors with gene-set enrichment analysis to possibly uncover more specific targets for therapeutic modulation. Although there was a remarkable decrease in the expression of genes in the rods and cones, we did not find any specific transcription factors associated with these genes. Instead, we found some enriched motifs associated with increased expression of genes in the rod cluster (such as STAT1 and STAT3). A STAT1 increase has been found through proteomic analysis in mouse RP model *Rd10* mice^47^. STAT3, activated by ciliary neurotrophic factor in retinal progenitor cells, regulates photoreceptor development in the early postnatal mouse retina^48,49^. We also observed other enriched motifs (such as IKZF1, Supplementary Table 9). IKZF1 regulates early temporal identity in retinal progenitors, and overexpression of IKZF1 in late mouse retinal progenitor cells increases the production of early-born cell types in the mouse retina^50^. It would be interesting to further explore the roles of these factors in mammalian retinal development and degeneration. We found that the JUND motif was enriched and associated with genes with increased expression in Müller glia/astrocytes. JUN regulates neuronal regenerative and degenerative responses after axonal injury^51^, and JUN activation is a key mediator controlling astrocytic gliosis^52^. Regulating JUND may modulate retinal glial responses and protect photoreceptors.

In conclusion, our findings show similar suppression of metabolic pathways in rods and cones accompanied by decreased expression of genes involved in photoreceptor maintenance. We have recently reported that activation of Müller glial remodeling preserves retinal function in mouse RP (P23H)^12^. Thus, modulating Müller glial compensatory responses might be a generalized approach to slow photoreceptor loss in RP and other retinal degenerative disorders.

## Supplementary information

Supplementary information.

## References

[1] Ali MU, Rahman MSU, Cao J, Yuan PX (2017). Genetic characterization and disease mechanism of retinitis pigmentosa; current scenario. 3 Biotech.

[2] Newton F, Megaw R (2020). Mechanisms of photoreceptor death in retinitis pigmentosa. Genes (Basel).

[3] Punzo C, Kornacker K, Cepko CL (2009). Stimulation of the insulin/mTOR pathway delays cone death in a mouse model of retinitis pigmentosa. Nat. Neurosci..

[4] Aït-Ali N (2015). Rod-derived cone viability factor promotes cone survival by stimulating aerobic glycolysis. Cell.

[5] Cheng SY (2020). Altered photoreceptor metabolism in mouse causes late stage age-related macular degeneration-like pathologies. Proc. Natl Acad. Sci. USA.

[6] Tam BM, Moritz OL (2006). Characterization of rhodopsin P23H-induced retinal degeneration in a *Xenopus laevis* model of retinitis pigmentosa. Invest. Ophthalmol. Vis. Sci..

[7] Sakami S (2011). Probing mechanisms of photoreceptor degeneration in a new mouse model of the common form of autosomal dominant retinitis pigmentosa due to P23H opsin mutations. J. Biol. Chem..

[8] Fu Z (2018). Fibroblast growth factor 21 protects photoreceptor function in type 1 diabetic mice. Diabetes.

[9] Zilionis R (2017). Single-cell barcoding and sequencing using droplet microfluidics. Nat. Protoc..

[10] Klein AM (2015). Droplet barcoding for single-cell transcriptomics applied to embryonic stem cells. Cell.

[11] Renthal W (2018). Characterization of human mosaic Rett syndrome brain tissue by single-nucleus RNA sequencing. Nat. Neurosci..

[12] Fu Z (2021). Retinal glial remodeling by FGF21 preserves retinal function during photoreceptor degeneration. iScience.

[13] Wolock SL, Lopez R, Klein AM (2019). Scrublet: computational identification of cell doublets in single-cell transcriptomic data. Cell Syst..

[14] Butler A, Hoffman P, Smibert P, Papalexi E, Satija R (2018). Integrating single-cell transcriptomic data across different conditions, technologies, and species. Nat. Biotechnol..

[15] Han X (2018). Mapping the mouse cell atlas by Microwell-seq. Cell.

[16] Robinson MD, McCarthy DJ, Smyth GK (2010). edgeR: a bioconductor package for differential expression analysis of digital gene expression data. Bioinformatics.

[17] Qiu C (2021). *Cis* P-tau underlies vascular contribution to cognitive impairment and dementia and can be effectively targeted by immunotherapy in mice. Sci. Transl. Med..

[18] Wanet A (2021). E-cadherin is regulated by GATA-2 and marks the early commitment of mouse hematopoietic progenitors to the basophil and mast cell fates. Sci. Immunol..

[19] Maroni G (2021). Identification of a targetable KRAS-mutant epithelial population in non-small cell lung cancer. Commun. Biol..

[20] Yu G, Wang LG, Han Y, He QY (2012). clusterProfiler: an R package for comparing biological themes among gene clusters. OMICS.

[21] Subramanian A (2005). Gene set enrichment analysis: a knowledge-based approach for interpreting genome-wide expression profiles. Proc. Natl. Acad. Sci. USA.

[22] Chen EY (2013). Enrichr: interactive and collaborative HTML5 gene list enrichment analysis tool. BMC Bioinformatics.

[23] Fu Z (2017). Photoreceptor glucose metabolism determines normal retinal vascular growth. EMBO Mol. Med..

[24] Fu Z (2017). Adiponectin mediates dietary omega-3 long-chain polyunsaturated fatty acid protection against choroidal neovascularization in mice. Invest. Ophthalmol. Vis. Sci..

[25] Lee ES, Lee JY, Jeon CJ (2010). Types and density of calretinin-containing retinal ganglion cells in mouse. Neurosci. Res..

[26] Haverkamp S, Wassle H (2000). Immunocytochemical analysis of the mouse retina. J. Comp. Neurol..

[27] Fu Z (2015). Deficiency of aldose reductase attenuates inner retinal neuronal changes in a mouse model of retinopathy of prematurity. Graefes Arch. Clin. Exp. Ophthalmol..

[28] Wubben TJ (2017). Photoreceptor metabolic reprogramming provides survival advantage in acute stress while causing chronic degeneration. Sci. Rep..

[29] Rajala A, Wang Y, Soni K, Rajala RVS (2018). Pyruvate kinase M2 isoform deletion in cone photoreceptors results in age-related cone degeneration. Cell Death Dis..

[30] Nangaku M (1994). KIF1B, a novel microtubule plus end-directed monomeric motor protein for transport of mitochondria. Cell.

[31] Jancsik V, Filliol D, Felter S, Rendon A (1989). Binding of microtubule-associated proteins (MAPs) to rat brain mitochondria: a comparative study of the binding of MAP2, its microtubule-binding and projection domains, and tau proteins. Cell Motil. Cytoskeleton.

[32] Jung D, Filliol D, Miehe M, Rendon A (1993). Interaction of brain mitochondria with microtubules reconstituted from brain tubulin and MAP2 or TAU. Cell Motil. Cytoskeleton.

[33] Joyal JS, Gantner ML, Smith LEH (2018). Retinal energy demands control vascular supply of the retina in development and disease: the role of neuronal lipid and glucose metabolism. Prog. Retin. Eye Res..

[34] Fu Z, Kern TS, Hellstrom A, Smith L (2020). Fatty acid oxidation and photoreceptor metabolic needs. J. Lipid Res..

[35] Kanow MA (2017). Biochemical adaptations of the retina and retinal pigment epithelium support a metabolic ecosystem in the vertebrate eye. eLife.

[36] Zhao C (2011). mTOR-mediated dedifferentiation of the retinal pigment epithelium initiates photoreceptor degeneration in mice. J. Clin. Invest..

[37] Goel M, Dhingra NK (2012). Muller glia express rhodopsin in a mouse model of inherited retinal degeneration. Neuroscience.

[38] Das SR, Bhardwaj N, Kjeldbye H, Gouras P (1992). Muller cells of chicken retina synthesize 11-*cis*-retinol. Biochem. J..

[39] Kaylor JJ (2014). Identification of the 11-*cis*-specific retinyl-ester synthase in retinal Muller cells as multifunctional O-acyltransferase (MFAT). Proc. Natl. Acad. Sci. USA.

[40] Goldman D (2014). Muller glial cell reprogramming and retina regeneration. Nat. Rev. Neurosci..

[41] Rueda EM (2019). The Hippo pathway blocks mammalian retinal Muller glial cell reprogramming. Cell Rep..

[42] Yao K (2018). Restoration of vision after de novo genesis of rod photoreceptors in mammalian retinas. Nature.

[43] Jorstad NL (2017). Stimulation of functional neuronal regeneration from Muller glia in adult mice. Nature.

[44] Pfeiffer RL, Marc RE, Kondo M, Terasaki H, Jones BW (2016). Muller cell metabolic chaos during retinal degeneration. Exp. Eye Res..

[45] Hayakawa K (2016). Transfer of mitochondria from astrocytes to neurons after stroke. Nature.

[46] Davis CH (2014). Transcellular degradation of axonal mitochondria. Proc. Natl. Acad. Sci. USA.

[47] Ly A (2016). Proteomic profiling suggests central role of STAT signaling during retinal degeneration in the rd10 mouse model. J. Proteome. Res..

[48] Rhee KD, Goureau O, Chen S, Yang XJ (2004). Cytokine-induced activation of signal transducer and activator of transcription in photoreceptor precursors regulates rod differentiation in the developing mouse retina. J. Neurosci..

[49] Zhang SS (2004). STAT3-mediated signaling in the determination of rod photoreceptor cell fate in mouse retina. Invest. Ophthalmol. Vis. Sci..

[50] Elliott J, Jolicoeur C, Ramamurthy V, Cayouette M (2008). Ikaros confers early temporal competence to mouse retinal progenitor cells. Neuron.

[51] Fernandes KA, Harder JM, Kim J, Libby RT (2013). JUN regulates early transcriptional responses to axonal injury in retinal ganglion cells. Exp. Eye Res..

[52] Gadea A, Schinelli S, Gallo V (2008). Endothelin-1 regulates astrocyte proliferation and reactive gliosis via a JNK/c-Jun signaling pathway. J. Neurosci..

