# Supplementary material for: Müller glial responses compensate for degenerating photoreceptors in retinitis pigmentosa
Source: Exp Mol Med. 2021 Nov 19;53(11):1748–58. doi: 10.1038/s12276-021-00693-w (PMC8639781; doi:10.1038/s12276-021-00693-w)
Supplement: Supplementary file 1 — Supplementary information. [file 12276_2021_693_MOESM1_ESM.pdf]

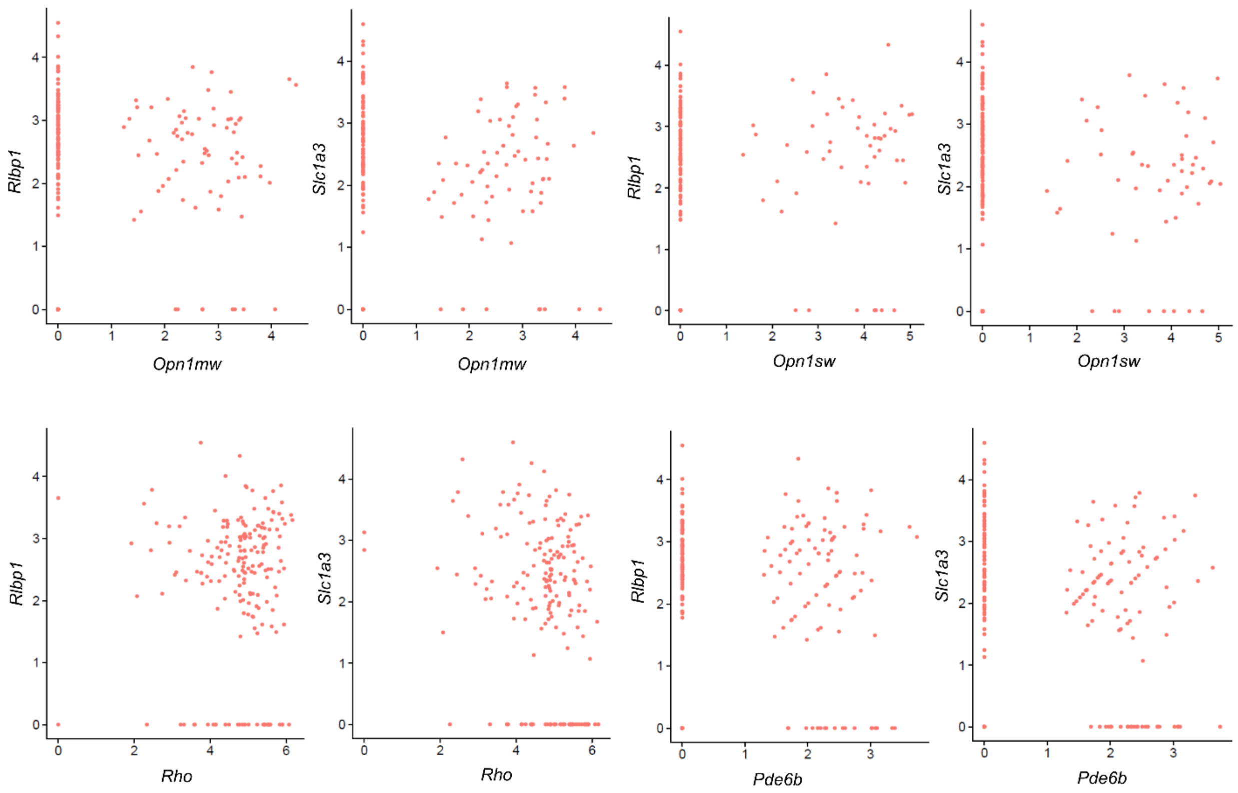

Supplementary Figure 1: X-Y scatter plots of normalized expression of marker genes for rod (*Rho, Pde6b*), cone (*Opn1mw, Opn1sw*) and Müller glial (*Rlbp1, Slc1a3*) in cluster 12 cells.

Supplementary Table 1, Cell number from each mouse sample.

Sample	Number of cells
WT_1	1651
WT_2	1539
WT_3	576
WT_4	419
Het_1	2031
Het_2	761
Het_3	880
Het_4	970

Supplementary Table 2, number of cells with >500 detectable genes in each cluster.

tSNE group	Cell type	Number of cells
0	Rods	1491
1	Bipolar_cells	1063
2	Rods	942
3	Cones	831
4	Bipolar_cells	772
5	Rods	702
6	Rods	647
7	Amacrine_cells	529
8	Amacrine_cells	517
9	Muller_glia	443
10	Muller_glia	398
11	Amacrine_cells	198
12	Mixed	19
13	Macrophage_Microglia	46
14	Endothelial_cells	34
15	Retinal_ganglion_cells	24

Supplementary Table 3, GO pathway analysis with downregulated genes in rods

ID	Description	GeneRatio	BgRatio	pvalue	p.adjust	qvalue	geneID	Count	GO_type	minus log10	gene rati
GO:0022626	cytosolic ribosome	33/268	111/18982	8.04E-35	2.69E-32	2.42E-32	Rpl18a/U	33	CC	34.09455	0.123
GO:0003735	structural constituent of ribosome	32/260	153/18634	1.32E-28	6.90E-26	6.30E-26	Rpl18a/R	32	MF	27.879034	0.123
GO:0044391	ribosomal subunit	33/268	190/18982	1.63E-26	2.14E-24	1.93E-24	Rpl18a/U	33	CC	25.787632	0.123
GO:0005840	ribosome	35/268	224/18982	1.92E-26	2.14E-24	1.93E-24	Rpl18a/U	35	CC	25.716661	0.131
GO:0007601	visual perception	27/264	157/18773	8.62E-22	1.91E-18	1.86E-18	Rho/Opn	27	BP	21.064243	0.102
GO:0001750	photoreceptor outer segment	21/268	76/18982	9.24E-22	7.71E-20	6.95E-20	Rho/Opn	21	CC	21.034545	0.078
GO:0050953	sensory perception of light stimulus	27/264	161/18773	1.72E-21	1.91E-18	1.86E-18	Rho/Opn	27	BP	20.763452	0.102
GO:0009583	detection of light stimulus	18/264	49/18773	2.02E-21	1.91E-18	1.86E-18	Rho/Opn	18	BP	20.694281	0.068
GO:0022627	cytosolic small ribosomal subunit	17/268	46/18982	2.56E-20	1.71E-18	1.54E-18	Uba52/Rp	17	CC	19.590945	0.063
GO:0097733	photoreceptor cell cilium	22/268	110/18982	2.09E-19	1.16E-17	1.05E-17	Rho/Opn	22	CC	18.680007	0.082
GO:0097731	9+0 non-motile cilium	22/268	116/18982	7.06E-19	3.37E-17	3.04E-17	Rho/Opn	22	CC	18.151017	0.082
GO:0022625	cytosolic large ribosomal subunit	17/268	60/18982	4.76E-18	1.99E-16	1.79E-16	Rpl18a/U	17	CC	17.322453	0.063
GO:0097730	non-motile cilium	23/268	148/18982	1.18E-17	4.38E-16	3.95E-16	Rho/Opn	23	CC	16.928268	0.086
GO:0015935	small ribosomal subunit	17/268	75/18982	3.02E-16	1.01E-14	9.09E-15	Uba52/Rp	17	CC	15.52002	0.063
GO:0007602	phototransduction	12/264	28/18773	1.16E-15	8.23E-13	8.00E-13	Rho/Opn	12	BP	14.935309	0.045
GO:0009582	detection of abiotic stimulus	19/264	127/18773	1.64E-14	8.99E-12	8.73E-12	Rho/Slc1	19	BP	13.78538	0.072
GO:0009581	detection of external stimulus	19/264	128/18773	1.90E-14	8.99E-12	8.73E-12	Rho/Slc1	19	BP	13.72103	0.072
GO:0042622	photoreceptor outer segment membrane	9/268	14/18982	3.67E-14	1.11E-12	1.00E-12	Rho/Pde6	9	CC	13.435381	0.034
GO:0002181	cytoplasmic translation	16/264	88/18773	8.70E-14	3.53E-11	3.43E-11	Rpl18a/Pl	16	BP	13.060424	0.061
GO:0009416	response to light stimulus	25/264	285/18773	3.24E-13	1.15E-10	1.12E-10	Rho/Opn	25	BP	12.489558	0.095
GO:0015934	large ribosomal subunit	17/268	120/18982	1.10E-12	3.05E-11	2.75E-11	Rpl18a/U	17	CC	11.95982	0.063
GO:0009584	detection of visible light	10/264	30/18773	5.99E-12	1.89E-09	1.83E-09	Rho/Prph	10	BP	11.222915	0.038
GO:0042461	photoreceptor cell development	12/264	59/18773	2.90E-11	8.23E-09	8.00E-09	Nr2e3/Nr	12	BP	10.537301	0.045
GO:0051606	detection of stimulus	20/264	236/18773	1.53E-10	3.95E-08	3.84E-08	Rho/Slc1	20	BP	9.8145251	0.076
GO:0009314	response to radiation	25/264	387/18773	2.56E-10	6.04E-08	5.87E-08	Rho/Opn	25	BP	9.59252	0.095
GO:0046530	photoreceptor cell differentiation	12/264	78/18773	8.89E-10	1.94E-07	1.88E-07	Nr2e3/Nr	12	BP	9.0513024	0.045
GO:0060041	retina development in camera-type eye	15/264	154/18773	4.81E-09	9.74E-07	9.47E-07	Rho/Nr2e	15	BP	8.3178647	0.057
GO:0000028	ribosomal small subunit assembly	7/264	20/18773	6.66E-09	1.26E-06	1.22E-06	Rps27/Rp	7	BP	8.1764447	0.027
GO:0001917	photoreceptor inner segment	10/268	59/18982	9.10E-09	2.34E-07	2.11E-07	Rho/Gnat	10	CC	8.0410304	0.037
GO:0060170	ciliary membrane	9/268	47/18982	1.66E-08	3.97E-07	3.57E-07	Rho/Pde6	9	CC	7.779326	0.034
GO:0042274	ribosomal small subunit biogenesis	10/264	69/18773	4.18E-08	7.42E-06	7.21E-06	Rps27/Rp	10	BP	7.3784149	0.038
GO:0005844	polysome	10/268	70/18982	5.01E-08	1.12E-06	1.01E-06	Rpl18a/R	10	CC	7.2999475	0.037
GO:0042788	polysomal ribosome	7/268	29/18982	1.24E-07	2.58E-06	2.33E-06	Rpl18a/R	7	CC	6.9074454	0.026
GO:0007603	phototransduction, visible light	5/264	10/18773	1.26E-07	2.05E-05	2.00E-05	Rho/Grk1	5	BP	6.8996639	0.019
GO:0045494	photoreceptor cell maintenance	8/264	43/18773	1.30E-07	2.05E-05	2.00E-05	Rho/Abca	8	BP	6.8850087	0.030
GO:0050908	detection of light stimulus involved in visual perception	6/264	20/18773	2.40E-07	3.40E-05	3.31E-05	Prph2/Ro	6	BP	6.6196716	0.023
GO:0050962	detection of light stimulus involved in sensory perception	6/264	20/18773	2.40E-07	3.40E-05	3.31E-05	Prph2/Ro	6	BP	6.6196716	0.023
GO:0001895	retina homeostasis	8/264	53/18773	7.05E-07	9.53E-05	9.26E-05	Rho/Abca	8	BP	6.151561	0.030
GO:0030507	spectrin binding	6/260	29/18634	2.53E-06	0.00065964	0.0006026	Rho/Pde6	6	MF	5.5973354	0.023
GO:0001654	eye development	19/264	387/18773	2.60E-06	0.00033489	0.0003255	Rho/Nr2e	19	BP	5.5853774	0.072
GO:0150063	visual system development	19/264	391/18773	3.02E-06	0.00036427	0.0003541	Rho/Nr2e	19	BP	5.5202772	0.072
GO:0042255	ribosome assembly	8/264	64/18773	3.08E-06	0.00036427	0.0003541	Rps27/Rp	8	BP	5.5110764	0.030
GO:0048880	sensory system development	19/264	395/18773	3.50E-06	0.0003969	0.0003858	Rho/Nr2e	19	BP	5.4560823	0.072
GO:0019843	rRNA binding	8/260	69/18634	5.17E-06	0.00089978	0.0008219	Rpl9/Rps	8	MF	5.2864127	0.031
GO:0046034	ATP metabolic process	14/264	233/18773	5.80E-06	0.00063299	0.0006153	Pgam1/Es	14	BP	5.2363368	0.053
GO:0043010	camera-type eye development	17/264	340/18773	6.95E-06	0.00073045	0.0007101	Rho/Nr2e	17	BP	5.1577519	0.064
GO:0071482	cellular response to light stimulus	9/264	95/18773	7.69E-06	0.00077841	0.0007567	Rho/Opn	9	BP	5.1143426	0.034
GO:0001754	eye photoreceptor cell differentiation	7/264	53/18773	8.91E-06	0.0008717	0.0008474	Nr2e3/Nr	7	BP	5.0499424	0.027
GO:0042462	eye photoreceptor cell development	6/264	39/18773	1.62E-05	0.00152672	0.0014841	Nr2e3/Nr	6	BP	4.7918263	0.023
GO:0004114	3',5'-cyclic-nucleotide phosphodiesterase activity	5/260	24/18634	1.74E-05	0.00196872	0.0017984	Pde6g/Pd	5	MF	4.7592209	0.019
GO:0008081	phosphoric diester hydrolase activity	8/260	82/18634	1.89E-05	0.00196872	0.0017984	Pde6g/Pd	8	MF	4.7245169	0.031
GO:0004112	cyclic-nucleotide phosphodiesterase activity	5/260	26/18634	2.63E-05	0.00229116	0.0020929	Pde6g/Pd	5	MF	4.5794647	0.019
GO:0035845	photoreceptor cell outer segment organization	4/264	14/18773	3.42E-05	0.00313314	0.0030457	Prph2/Ro	4	BP	4.4653647	0.015
GO:0047555	3',5'-cyclic-GMP phosphodiesterase activity	4/260	15/18634	4.48E-05	0.00334004	0.0030511	Pde6g/Pd	4	MF	4.3488209	0.015
GO:0042254	ribosome biogenesis	14/264	283/18773	5.08E-05	0.00450602	0.0043803	Rps27/Rp	14	BP	4.2937629	0.053
GO:0031253	cell projection membrane	14/268	293/18982	7.72E-05	0.00151645	0.0013669	Rho/Kcnb	14	CC	4.1124684	0.052
GO:0043209	myelin sheath	11/268	205/18982	0.0001662	0.00308399	0.0027798	Pgam1/G	11	CC	3.779361	0.041

Supplementary Table 4, GO pathway analysis with upregulated genes in rods

ID	Description	GeneRatio	BgRatio	pvalue	p.adjust	qvalue	geneID	Count	GO_type	minus log10	GeneRatio
GO:0000979	RNA polymerase II core promoter sequence-specific DI	5/156	22/18634	9.05E-07	0.00019757	0.00018436	Stat1/Gtf2a1/Fos/Egr1/H3f3a	5	MF	6.04324749	0.03205128
GO:0031720	haptoglobin binding	4/156	10/18634	9.54E-07	0.00019757	0.00018436	Hbb-bt/Hba-a1/Hba-a2/Hbb-bs	4	MF	6.02025081	0.02564103
GO:0005833	hemoglobin complex	4/162	10/18982	1.03E-06	0.00016691	0.00015253	Hbb-bt/Hba-a1/Hba-a2/Hbb-bs	4	CC	5.98655634	0.02469136
GO:0043209	myelin sheath	11/162	205/18982	1.54E-06	0.00016691	0.00015253	Ubb/Nefh/Ckb/Eef1a1/Fscn1/Nme2/Ppi	11	CC	5.81357775	0.06790123
GO:0031838	haptoglobin-hemoglobin complex	4/162	11/18982	1.61E-06	0.00016691	0.00015253	Hbb-bt/Hba-a1/Hba-a2/Hbb-bs	4	CC	5.79315979	0.02469136
GO:0019825	oxygen binding	4/156	23/18634	3.70E-05	0.00461025	0.00430196	Hbb-bt/Hba-a1/Hba-a2/Hbb-bs	4	MF	4.43215008	0.02564103
GO:0001046	core promoter sequence-specific DNA binding	5/156	47/18634	4.45E-05	0.00461025	0.00430196	Stat1/Gtf2a1/Fos/Egr1/H3f3a	5	MF	4.35121571	0.03205128
GO:0097440	apical dendrite	4/162	28/18982	8.92E-05	0.0069357	0.00633826	Slc1a1/Nefh/Neurl1a/Clu	4	CC	4.04961009	0.02469136

Supplementary Table 5, GO pathway analysis with downregulated genes in cones

ID	Description	GeneRatio	BgRatio	pvalue	p.adjust	qvalue	geneID	GO_type	minus log10	GeneRatio
GO:0042622	photoreceptor outer segment membrane	10/112	14/18982	3.33E-20	7.02E-18	5.78E-18	Rho/Gnb1/Cnga1/Pde6a/Pde6b/Rom1/Pde6g/Cdhr1/Prcd/Pde6h	CC	19.4779339	0.08928571
GO:0001750	photoreceptor outer segment	14/112	76/18982	1.36E-17	1.43E-15	1.18E-15	Rho/Gnat1/Gnb1/Cnga1/Pde6a/Prph2/Pde6b/Rom1/Pde6g/Cdhr1/Prcd/Gngt1/Pde6h/Gna	CC	16.8667605	0.125
GO:0007601	visual perception	17/107	157/18773	2.41E-17	2.61E-14	2.39E-14	Rho/Gnat1/Reep6/Cnga1/Pde6a/Prph2/Pde6b/Rom1/Pde6g/Rcvrn/Unc119/Prcd/Rbp3/Cak	BP	16.617949	0.1588785
GO:0050953	sensory perception of light stimulus	17/107	161/18773	3.71E-17	2.61E-14	2.39E-14	Rho/Gnat1/Reep6/Cnga1/Pde6a/Prph2/Pde6b/Rom1/Pde6g/Rcvrn/Unc119/Prcd/Rbp3/Cak	BP	16.4300858	0.1588785
GO:0009583	detection of light stimulus	12/107	49/18773	4.82E-17	2.61E-14	2.39E-14	Rho/Gnat1/Gnb1/Reep6/Prph2/Pde6b/Rom1/Rcvrn/Cabp4/Gngt1/Aipl1/Gnat2	BP	16.3165846	0.11214953
GO:0097733	photoreceptor cell cilium	14/112	110/18982	3.08E-15	2.17E-13	1.78E-13	Rho/Gnat1/Gnb1/Cnga1/Pde6a/Prph2/Pde6b/Rom1/Pde6g/Cdhr1/Prcd/Gngt1/Pde6h/Gna	CC	14.5112719	0.125
GO:0097731	9+0 non-motile cilium	14/112	116/18982	6.60E-15	3.48E-13	2.86E-13	Rho/Gnat1/Gnb1/Cnga1/Pde6a/Prph2/Pde6b/Rom1/Pde6g/Cdhr1/Prcd/Gngt1/Pde6h/Gna	CC	14.1807759	0.125
GO:0022626	cytosolic ribosome	13/112	111/18982	9.54E-14	4.02E-12	3.31E-12	Rpl23a/Rps18/Rpl6/Rps13/Rpl21/Rpl18a/Rps2/Rpl35a/Rps29/Rps27/Rps25/Rplp1/Rps14	CC	13.020614	0.11607143
GO:0060170	ciliary membrane	10/112	47/18982	1.46E-13	5.15E-12	4.24E-12	Rho/Gnb1/Cnga1/Pde6a/Pde6b/Rom1/Pde6g/Cdhr1/Prcd/Pde6h	CC	12.8344294	0.08928571
GO:0097730	non-motile cilium	14/112	148/18982	2.05E-13	6.19E-12	5.09E-12	Rho/Gnat1/Gnb1/Cnga1/Pde6a/Prph2/Pde6b/Rom1/Pde6g/Cdhr1/Prcd/Gngt1/Pde6h/Gna	CC	12.6877187	0.125
GO:0009582	detection of abiotic stimulus	13/107	127/18773	3.52E-13	1.26E-10	1.16E-10	Rho/Gnat1/Gnb1/Reep6/Prph2/Pde6b/Rom1/Slc12a2/Rcvrn/Cabp4/Gngt1/Aipl1/Gnat2	BP	12.4538293	0.12149533
GO:0009581	detection of external stimulus	13/107	128/18773	3.90E-13	1.26E-10	1.16E-10	Rho/Gnat1/Gnb1/Reep6/Prph2/Pde6b/Rom1/Slc12a2/Rcvrn/Cabp4/Gngt1/Aipl1/Gnat2	BP	12.4093483	0.12149533
GO:0007602	phototransduction	8/107	28/18773	2.41E-12	6.53E-10	5.99E-10	Rho/Gnat1/Gnb1/Rcvrn/Cabp4/Gngt1/Aipl1/Gnat2	BP	11.6172795	0.07476636
GO:0009584	detection of visible light	8/107	30/18773	4.50E-12	1.04E-09	9.58E-10	Rho/Gnat1/Gnb1/Reep6/Prph2/Rom1/Aipl1/Gnat2	BP	11.3464841	0.07476636
GO:0003735	structural constituent of ribosome	13/108	153/18634	4.85E-12	1.42E-09	1.16E-09	Rpl23a/Rps18/Rpl6/Rps13/Rpl21/Rpl18a/Rps2/Rpl35a/Rps29/Rps27/Rps25/Rplp1/Rps14	MF	11.3143703	0.12037037
GO:0042461	photoreceptor cell development	9/107	59/18773	4.47E-11	9.07E-09	8.31E-09	Gnat1/Nrl/Nr2e3/Prph2/Rom1/Cdhr1/Cabp4/Gngt1/Gnat2	BP	10.3497889	0.08411215
GO:0001917	photoreceptor inner segment	9/112	59/18982	6.14E-11	1.62E-09	1.33E-09	Rho/Gnat1/Gnb1/Reep6/Pkm/Guk1/Gngt1/Aipl1/Gnat2	CC	10.2117215	0.08035714
GO:0044391	ribosomal subunit	13/112	190/18982	9.59E-11	2.25E-09	1.85E-09	Rpl23a/Rps18/Rpl6/Rps13/Rpl21/Rpl18a/Rps2/Rpl35a/Rps29/Rps27/Rps25/Rplp1/Rps14	CC	10.0182248	0.11607143
GO:0009135	purine nucleoside diphosphate metabolic process	10/107	100/18773	2.66E-10	4.32E-08	3.96E-08	Aldoa/Esrrb/Pkm/Pfkl/Pgam1/Tpi1/Guk1/Eno1/Eno1b/Mif	BP	9.57465922	0.09345794
GO:0009179	purine ribonucleoside diphosphate metabolic process	10/107	100/18773	2.66E-10	4.32E-08	3.96E-08	Aldoa/Esrrb/Pkm/Pfkl/Pgam1/Tpi1/Guk1/Eno1/Eno1b/Mif	BP	9.57465922	0.09345794
GO:0046939	nucleotide phosphorylation	10/107	103/18773	3.58E-10	5.28E-08	4.84E-08	Aldoa/Esrrb/Pkm/Pfkl/Pgam1/Tpi1/Guk1/Eno1/Eno1b/Mif	BP	9.44635479	0.09345794
GO:0009185	ribonucleoside diphosphate metabolic process	10/107	104/18773	3.94E-10	5.33E-08	4.89E-08	Aldoa/Esrrb/Pkm/Pfkl/Pgam1/Tpi1/Guk1/Eno1/Eno1b/Mif	BP	9.40449672	0.09345794
GO:0046530	photoreceptor cell differentiation	9/107	78/18773	5.93E-10	7.40E-08	6.79E-08	Gnat1/Nrl/Nr2e3/Prph2/Rom1/Cdhr1/Cabp4/Gngt1/Gnat2	BP	9.22691073	0.08411215
GO:0005840	ribosome	13/112	224/18982	7.37E-10	1.55E-08	1.28E-08	Rpl23a/Rps18/Rpl6/Rps13/Rpl21/Rpl18a/Rps2/Rpl35a/Rps29/Rps27/Rps25/Rplp1/Rps14	CC	9.13263354	0.11607143
GO:0051606	detection of stimulus	13/107	236/18773	8.97E-10	1.04E-07	9.53E-08	Rho/Gnat1/Gnb1/Reep6/Prph2/Pde6b/Rom1/Slc12a2/Rcvrn/Cabp4/Gngt1/Aipl1/Gnat2	BP	9.04729674	0.12149533
GO:0060041	retina development in camera-type eye	11/107	154/18773	1.25E-09	1.35E-07	1.24E-07	Rho/Gnat1/Nrl/Gnb1/Nr2e3/Pde6a/Prph2/Pde6b/Rom1/Cabp4/Gnat2	BP	8.90476349	0.10280374
GO:0009132	nucleoside diphosphate metabolic process	10/107	118/18773	1.38E-09	1.40E-07	1.28E-07	Aldoa/Esrrb/Pkm/Pfkl/Pgam1/Tpi1/Guk1/Eno1/Eno1b/Mif	BP	8.8610147	0.09345794
GO:0006096	glycolytic process	9/107	89/18773	1.96E-09	1.77E-07	1.62E-07	Aldoa/Esrrb/Pkm/Pfkl/Pgam1/Tpi1/Eno1/Eno1b/Mif	BP	8.70715574	0.08411215
GO:0006757	ATP generation from ADP	9/107	89/18773	1.96E-09	1.77E-07	1.62E-07	Aldoa/Esrrb/Pkm/Pfkl/Pgam1/Tpi1/Eno1/Eno1b/Mif	BP	8.70715574	0.08411215
GO:0046031	ADP metabolic process	9/107	95/18773	3.53E-09	3.01E-07	2.76E-07	Aldoa/Esrrb/Pkm/Pfkl/Pgam1/Tpi1/Eno1/Eno1b/Mif	BP	8.45259946	0.08411215
GO:0006165	nucleoside diphosphate phosphorylation	9/107	101/18773	6.09E-09	4.95E-07	4.54E-07	Aldoa/Esrrb/Pkm/Pfkl/Pgam1/Tpi1/Eno1/Eno1b/Mif	BP	8.21511352	0.08411215
GO:0009416	response to light stimulus	13/107	285/18773	8.78E-09	6.79E-07	6.22E-07	Rho/Gnat1/Gnb1/Reep6/Prph2/Pde6b/Rom1/Rcvrn/Stk11/Cabp4/Gngt1/Aipl1/Gnat2	BP	8.05649226	0.12149533
GO:0022627	cytosolic small ribosomal subunit	7/112	46/18982	9.12E-09	1.75E-07	1.44E-07	Rps18/Rps13/Rps2/Rps29/Rps27/Rps25/Rps14	CC	8.04004517	0.0625
GO:0001754	eye photoreceptor cell differentiation	7/107	53/18773	1.99E-08	1.47E-06	1.35E-06	Gnat1/Nrl/Nr2e3/Rom1/Cabp4/Gngt1/Gnat2	BP	7.70096249	0.06542056
GO:0006090	pyruvate metabolic process	9/107	117/18773	2.23E-08	1.58E-06	1.45E-06	Aldoa/Esrrb/Pkm/Pfkl/Pgam1/Tpi1/Eno1/Eno1b/Mif	BP	7.65072316	0.08411215
GO:0002181	cytoplasmic translation	8/107	88/18773	3.77E-08	2.55E-06	2.34E-06	Pkm/Tma7/Rpl6/Rpl18a/Rps2/Rpl35a/Rps29/Rplp1	BP	7.42413121	0.07476636
GO:0050908	detection of light stimulus involved in visual perception	5/107	20/18773	7.93E-08	4.95E-06	4.54E-06	Gnat1/Reep6/Prph2/Rom1/Gnat2	BP	7.10079686	0.04672897
GO:0050962	detection of light stimulus involved in sensory perceptio	5/107	20/18773	7.93E-08	4.95E-06	4.54E-06	Gnat1/Reep6/Prph2/Rom1/Gnat2	BP	7.10079686	0.04672897
GO:0042462	eye photoreceptor cell development	6/107	39/18773	8.33E-08	5.01E-06	4.59E-06	Gnat1/Nrl/Nr2e3/Cabp4/Gngt1/Gnat2	BP	7.07914885	0.05607477
GO:0046034	ATP metabolic process	11/107	233/18773	9.26E-08	5.36E-06	4.92E-06	Aldoa/Esrrb/Pkm/Pfkl/Pgam1/Tpi1/Guk1/Uqcrh/Eno1/Eno1b/Mif	BP	7.03361957	0.10280374
GO:0016052	carbohydrate catabolic process	9/107	139/18773	1.00E-07	5.59E-06	5.13E-06	Aldoa/Esrrb/Pkm/Pfkl/Pgam1/Tpi1/Eno1/Eno1b/Mif	BP	7.00012639	0.08411215
GO:0007603	phototransduction, visible light	4/107	10/18773	2.04E-07	1.10E-05	1.01E-05	Rho/Gnat1/Gnb1/Aipl1	BP	6.69032539	0.03738318
GO:0015935	small ribosomal subunit	7/112	75/18982	2.94E-07	5.17E-06	4.25E-06	Rps18/Rps13/Rps2/Rps29/Rps27/Rps25/Rps14	CC	6.53189975	0.0625
GO:0001654	eye development	13/107	387/18773	3.12E-07	1.58E-05	1.45E-05	Rho/Gnat1/Nrl/Gnb1/Nr2e3/Pde6a/Prph2/Pde6b/Rom1/Cabp4/Gngt1/Sh3pxd2b/Gnat2	BP	6.50521572	0.12149533
GO:0009314	response to radiation	13/107	387/18773	3.12E-07	1.58E-05	1.45E-05	Rho/Gnat1/Gnb1/Reep6/Prph2/Pde6b/Rom1/Rcvrn/Stk11/Cabp4/Gngt1/Aipl1/Gnat2	BP	6.50521572	0.12149533
GO:0150063	visual system development	13/107	391/18773	3.51E-07	1.73E-05	1.58E-05	Rho/Gnat1/Nrl/Gnb1/Nr2e3/Pde6a/Prph2/Pde6b/Rom1/Cabp4/Gngt1/Sh3pxd2b/Gnat2	BP	6.45441567	0.12149533
GO:0048880	sensory system development	13/107	395/18773	3.94E-07	1.88E-05	1.73E-05	Rho/Gnat1/Nrl/Gnb1/Nr2e3/Pde6a/Prph2/Pde6b/Rom1/Cabp4/Gngt1/Sh3pxd2b/Gnat2	BP	6.40422588	0.12149533
GO:0031253	cell projection membrane	11/112	293/18982	1.30E-06	2.10E-05	1.73E-05	Rho/Gnb1/Cnga1/Pde6a/Pde6b/Rom1/Pde6g/Kcnb1/Cdhr1/Prcd/Pde6h	CC	5.887634	0.09821429
GO:0047555	3',5'-cyclic-GMP phosphodiesterase activity	4/108	15/18634	1.39E-06	0.00020246	0.0001664	Pde6a/Pde6b/Pde6g/Pde6h	MF	5.85801638	0.03703704
GO:0022625	cytosolic large ribosomal subunit	6/112	60/18982	1.42E-06	2.15E-05	1.77E-05	Rpl23a/Rpl6/Rpl21/Rpl18a/Rpl35a/Rplp1	CC	5.84640632	0.05357143
GO:0042255	ribosome assembly	6/107	64/18773	1.71E-06	7.91E-05	7.26E-05	Rpl23a/Rpl6/Rps2/Rps27/Rps25/Rps14	BP	5.76788494	0.05607477
GO:0019843	rRNA binding	6/108	69/18634	2.94E-06	0.00028588	0.00023497	Rpl23a/Rps18/Rpl6/Rps13/Ncl/Rps14	MF	5.53207832	0.05555556
GO:0043010	camera-type eye development	11/107	340/18773	3.85E-06	0.00017363	0.00015924	Rho/Gnat1/Nrl/Gnb1/Nr2e3/Pde6a/Prph2/Pde6b/Rom1/Cabp4/Gnat2	BP	5.41438429	0.10280374
GO:0000028	ribosomal small subunit assembly	4/107	20/18773	4.50E-06	0.0001976	0.00018121	Rps2/Rps27/Rps25/Rps14	BP	5.34633111	0.03738318
GO:0022618	ribonucleoprotein complex assembly	8/107	166/18773	4.90E-06	0.0002091	0.00019176	Rpl23a/Rpl6/Prpf8/Rps2/Rps27/Rps25/Rps14/Isy1	BP	5.31018479	0.07476636
GO:0071826	ribonucleoprotein complex subunit organization	8/107	173/18773	6.64E-06	0.0002763	0.00025339	Rpl23a/Rpl6/Prpf8/Rps2/Rps27/Rps25/Rps14/Isy1	BP	5.17788026	0.07476636
GO:0004114	3',5'-cyclic-nucleotide phosphodiesterase activity	4/108	24/18634	1.04E-05	0.00075698	0.00062218	Pde6a/Pde6b/Pde6g/Pde6h	MF	4.98423619	0.03703704
GO:0009150	purine ribonucleotide metabolic process	10/107	311/18773	1.15E-05	0.00046502	0.00042646	Aldoa/Esrrb/Pkm/Pfkl/Pgam1/Tpi1/Guk1/Eno1/Eno1b/Mif	BP	4.94078789	0.09345794
GO:0001895	retina homeostasis	5/107	53/18773	1.26E-05	0.00050032	0.00045883	Rho/Pde6a/Esrrb/Cdhr1/Aipl1	BP	4.89828657	0.04672897
GO:0050906	detection of stimulus involved in sensory perception	7/107	137/18773	1.34E-05	0.00051893	0.0004759	Rho/Gnat1/Reep6/Prph2/Rom1/Slc12a2/Gnat2	BP	4.87196056	0.06542056
GO:0004112	cyclic-nucleotide phosphodiesterase activity	4/108	26/18634	1.45E-05	0.00084445	0.00069407	Pde6a/Pde6b/Pde6g/Pde6h	MF	4.83984101	0.03703704
GO:0009259	ribonucleotide metabolic process	10/107	321/18773	1.51E-05	0.00056888	0.00052171	Aldoa/Esrrb/Pkm/Pfkl/Pgam1/Tpi1/Guk1/Eno1/Eno1b/Mif	BP	4.82182823	0.09345794
GO:0019693	ribose phosphate metabolic process	10/107	332/18773	2.01E-05	0.00074284	0.00068124	Aldoa/Esrrb/Pkm/Pfkl/Pgam1/Tpi1/Guk1/Eno1/Eno1b/Mif	BP	4.69597302	0.09345794
GO:0006163	purine nucleotide metabolic process	10/107	339/18773	2.41E-05	0.00086826	0.00079626	Aldoa/Esrrb/Pkm/Pfkl/Pgam1/Tpi1/Guk1/Eno1/Eno1b/Mif	BP	4.61845644	0.09345794
GO:0060219	camera-type eye photoreceptor cell differentiation	4/107	33/18773	3.59E-05	0.00126797	0.00116283	Nrl/Rom1/Cabp4/Gnat2	BP	4.44445265	0.03738318
GO:0005834	heterotrimeric G-protein complex	4/112	33/18982	4.12E-05	0.0005431	0.00044705	Gnat1/Gnb1/Gngt1/Gnat2	CC	4.38528416	0.03571429
GO:1905360	GTPase complex	4/112	33/18982	4.12E-05	0.0005431	0.00044705	Gnat1/Gnb1/Gngt1/Gnat2	CC	4.38528416	0.03571429
GO:0019318	hexose metabolic process	8/107	224/18773	4.29E-05	0.00148114	0.00135832	Aldoa/Fam3c/Esrrb/Pkm/Pfkl/Tpi1/G6pc3/Stk11	BP	4.36762462	0.07476636
GO:0006091	generation of precursor metabolites and energy	10/107	375/18773	5.65E-05	0.00190913	0.00175083	Aldoa/Esrrb/Pkm/Pfkl/Pgam1/Tpi1/Uqcrh/Eno1/Eno1b/Mif	BP	4.24824145	0.09345794
GO:0048592	eye morphogenesis	7/107	174/18773	6.23E-05	0.00203202	0.00186352	Gnat1/Nrl/Nr2e3/Rom1/Cabp4/Gngt1/Gnat2	BP	4.20532457	0.06542056
GO:0035845	photoreceptor cell outer segment organization	3/107	14/18773	6.26E-05	0.00203202	0.00186352	Prph2/Rom1/Cdhr1	BP	4.20342123	0.02803738
GO:0072521	purine-containing compound metabolic process	10/107	381/18773	6.45E-05	0.00205112	0.00188104	Aldoa/Esrrb/Pkm/Pfkl/Pgam1/Tpi1/Guk1/Eno1/Eno1b/Mif	BP	4.19075781	0.09345794
GO:0015934	large ribosomal subunit	6/112	120/18982	7.80E-05	0.00096845	0.00079718	Rpl23a/Rpl6/Rpl21/Rpl18a/Rpl35a/Rplp1	CC	4.10775717	0.05357143
GO:0022613	ribonucleoprotein complex biogenesis	10/107	392/18773	8.16E-05	0.00254744	0.00233621	Rpl23a/Rpl6/Ncl/Prpf8/Rps2/Rpl35a/Rps27/Rps25/Rps14/Isy1	BP	4.08821082	0.09345794
GO:0030553	cGMP binding	3/108	15/18634	8.19E-05	0.00398531	0.0032756	Cnga1/Pde6g/Pde6h	MF	4.08676915	0.02777778
GO:0005996	monosaccharide metabolic process	8/107	247/18773	8.52E-05	0.00260862	0.00239231	Aldoa/Fam3c/Esrrb/Pkm/Pfkl/Tpi1/G6pc3/Stk11	BP	4.06963188	0.07476636
GO:0045494	photoreceptor cell maintenance	4/107	43/18773	0.00010374	0.00311796	0.00285942	Rho/Pde6a/Esrrb/Cdhr1	BP	3.98405472	0.03738318
GO:0008081	phosphoric diester hydrolase activity	5/108	82/18634	0.00011404	0.00475717	0.00391	Gnb1/Pde6a/Pde6b/Pde6g/Pde6h	MF	3.94293639	0.0462963
GO:0009117	nucleotide metabolic process	10/107	411/18773	0.00012044	0.00355416	0.00325945	Aldoa/Esrrb/Pkm/Pfkl/Pgam1/Tpi1/Guk1/Eno1/Eno1b/Mif	BP	3.9192191	0.09345794
GO:0006006	glucose metabolic process	7/107	195/18773	0.0001272	0.00368653	0.00338084	Fam3c/Esrrb/Pkm/Pfkl/Tpi1/G6pc3/Stk11	BP	3.89551266	0.06542056
GO:0006753	nucleoside phosphate metabolic process	10/107	419/18773	0.00014094	0.00401317	0.0036804	Aldoa/Esrrb/Pkm/Pfkl/Pgam1/Tpi1/Guk1/Eno1/Eno1b/Mif	BP	3.85095588	0.09345794
GO:0042254	ribosome biogenesis	8/107	283/18773	0.00021693	0.00607043	0.00556707	Rpl23a/Rpl6/Ncl/Rps2/Rpl35a/Rps27/Rps25/Rps14	BP	3.66367088	0.07476636
GO:0003729	mRNA binding	8/108	286/18634	0.000261	0.00952652	0.00783002	Pkm/Rpl6/Rps13/Ncl/Pcbp4/Hnrnpu/Rps2/Rps14	MF	3.58335849	0.07407407
GO:0070069	cytochrome complex	3/112	26/18982	0.00047092	0.00552026	0.00454399	Uqcrh/Cox8a/Ndufa4	CC	3.32705054	0.02678571
GO:0042788	polysomal ribosome	3/112	29/18982	0.00065336	0.00714515	0.00588152	Rpl6/Rpl18a/Rps29	CC	3.18484566	0.02678571
GO:0019897	extrinsic component of plasma membrane	6/112	179/18982	0.00067727	0.00714515	0.00588152	Gnat1/Gnb1/Eno1/Gngt1/Rtbdn/Gnat2	CC	3.16924082	0.05357143
GO:0005844	polysome	4/112	70/18982	0.00078037	0.0078409	0.00645422	Rpl6/Rpl18a/Rps29/Rps25	CC	3.10769729	0.03571429

Supplementary Table 6, GO pathway analysis with upregulated genes in Müller glia

ID	Description	GeneRatio	BgRatio	pvalue	p.adjust	qvalue	geneID	Count	GO_type	minus log10	GeneRatio
GO:0001750	photoreceptor outer segment	20/214	76/18982	2.80E-22	1.03E-19	8.16E-20	Gucy2e/Tulp1/P	20	CC	21.5529657	0.09345794
GO:0007601	visual perception	25/210	157/18773	7.25E-22	2.13E-18	1.81E-18	Gucy2e/Nxnl2/T	25	BP	21.1397282	0.11904762
GO:0050953	sensory perception of light stimulus	25/210	161/18773	1.38E-21	2.13E-18	1.81E-18	Gucy2e/Nxnl2/T	25	BP	20.8601999	0.11904762
GO:0097733	photoreceptor cell cilium	21/214	110/18982	3.59E-20	6.60E-18	5.23E-18	Gucy2e/Tulp1/P	21	CC	19.4451618	0.09813084
GO:0097731	9+0 non-motile cilium	21/214	116/18982	1.16E-19	1.42E-17	1.12E-17	Gucy2e/Tulp1/P	21	CC	18.9373981	0.09813084
GO:0097730	non-motile cilium	21/214	148/18982	2.18E-17	2.01E-15	1.59E-15	Gucy2e/Tulp1/P	21	CC	16.661359	0.09813084
GO:0042622	photoreceptor outer segment membrane	9/214	14/18982	4.74E-15	3.49E-13	2.76E-13	Gucy2e/Phlpp2/	9	CC	14.324329	0.04205607
GO:0060041	retina development in camera-type eye	17/210	154/18773	1.58E-12	1.63E-09	1.38E-09	Tulp1/Hif1a/Rpg	17	BP	11.8011483	0.08095238
GO:0001917	photoreceptor inner segment	12/214	59/18982	2.17E-12	1.33E-10	1.06E-10	Tulp1/Phlpp2/R	12	CC	11.662589	0.05607477
GO:0048592	eye morphogenesis	16/210	174/18773	1.19E-10	8.60E-08	7.29E-08	Tulp1/Bdnf/Hif1	16	BP	9.92539873	0.07619048
GO:0009583	detection of light stimulus	10/210	49/18773	1.39E-10	8.60E-08	7.29E-08	Tulp1/Rom1/Rs	10	BP	9.85666562	0.04761905
GO:0001754	eye photoreceptor cell differentiation	10/210	53/18773	3.17E-10	1.64E-07	1.39E-07	Tulp1/Rpgrip1/(	10	BP	9.49829929	0.04761905
GO:0007602	phototransduction	8/210	28/18773	5.50E-10	2.43E-07	2.06E-07	Rs1/Gnb1/Gngt	8	BP	9.25943262	0.03809524
GO:0060170	ciliary membrane	9/214	47/18982	2.34E-09	1.23E-07	9.74E-08	Gucy2e/Phlpp2/	9	CC	8.63129989	0.04205607
GO:0001654	eye development	21/210	387/18773	2.67E-09	1.03E-06	8.76E-07	Tulp1/Bdnf/Hif1	21	BP	8.57295479	0.1
GO:0150063	visual system development	21/210	391/18773	3.21E-09	1.10E-06	9.35E-07	Tulp1/Bdnf/Hif1	21	BP	8.49363691	0.1
GO:0048880	sensory system development	21/210	395/18773	3.84E-09	1.19E-06	1.01E-06	Tulp1/Bdnf/Hif1	21	BP	8.41532662	0.1
GO:0003407	neural retina development	10/210	70/18773	5.48E-09	1.54E-06	1.31E-06	Rpgrip1/Casz1/F	10	BP	8.26160591	0.04761905
GO:0001895	retina homeostasis	9/210	53/18773	6.69E-09	1.72E-06	1.46E-06	Nxnl2/Tulp1/Pc	9	BP	8.17482081	0.04285714
GO:0090596	sensory organ morphogenesis	18/210	301/18773	8.62E-09	2.05E-06	1.74E-06	Tulp1/Pcdh15/B	18	BP	8.06432199	0.08571429
GO:0043010	camera-type eye development	19/210	340/18773	9.87E-09	2.18E-06	1.85E-06	Tulp1/Hif1a/Rpg	19	BP	8.00552673	0.09047619
GO:0046530	photoreceptor cell differentiation	10/210	78/18773	1.61E-08	3.31E-06	2.81E-06	Tulp1/Rpgrip1/(	10	BP	7.79403529	0.04761905
GO:0009582	detection of abiotic stimulus	12/210	127/18773	1.95E-08	3.77E-06	3.20E-06	Prrxl1/Tulp1/Pc	12	BP	7.70941841	0.05714286
GO:0009581	detection of external stimulus	12/210	128/18773	2.13E-08	3.82E-06	3.24E-06	Prrxl1/Tulp1/Pc	12	BP	7.67091113	0.05714286
GO:0045494	photoreceptor cell maintenance	8/210	43/18773	2.22E-08	3.82E-06	3.24E-06	Nxnl2/Tulp1/Pc	8	BP	7.65304036	0.03809524
GO:0060219	camera-type eye photoreceptor cell differentiation	7/210	33/18773	6.62E-08	1.08E-05	9.13E-06	Rpgrip1/Casz1/F	7	BP	7.17920686	0.03333333
GO:0060042	retina morphogenesis in camera-type eye	9/210	72/18773	1.07E-07	1.65E-05	1.40E-05	Rpgrip1/Casz1/F	9	BP	6.97109325	0.04285714
GO:0031253	cell projection membrane	16/214	293/18982	2.32E-07	1.07E-05	8.44E-06	Pdpn/Gucy2e/N	16	CC	6.63529669	0.07476636
GO:0048593	camera-type eye morphogenesis	11/210	143/18773	6.28E-07	9.25E-05	7.85E-05	Hif1a/Rpgrip1/C	11	BP	6.20182066	0.05238095
GO:0030507	spectrin binding	6/207	29/18634	6.71E-07	0.00029048	0.00025069	Myo10/Epb41/F	6	MF	6.17336542	0.02898551
GO:0009584	detection of visible light	6/210	30/18773	8.66E-07	0.00012168	0.00010318	Tulp1/Rom1/Rs	6	BP	6.06261092	0.02857143
GO:0051606	detection of stimulus	13/210	236/18773	2.69E-06	0.00036052	0.00030573	Prrxl1/Tulp1/Pc	13	BP	5.57079543	0.06190476
GO:0034643	establishment of mitochondrion localization, microtubule-mediated	5/210	21/18773	2.94E-06	0.00036052	0.00030573	Ubb/Kif1b/Hif1a	5	BP	5.53206184	0.02380952
GO:0047497	mitochondrion transport along microtubule	5/210	21/18773	2.94E-06	0.00036052	0.00030573	Ubb/Kif1b/Hif1a	5	BP	5.53206184	0.02380952
GO:0097499	protein localization to non-motile cilium	4/210	10/18773	3.03E-06	0.00036052	0.00030573	Tulp1/Rom1/Arl	4	BP	5.51833936	0.01904762
GO:0042461	photoreceptor cell development	7/210	59/18773	4.13E-06	0.00046506	0.00039438	Tulp1/Rpgrip1/F	7	BP	5.38382873	0.03333333
GO:0009416	response to light stimulus	14/210	285/18773	4.21E-06	0.00046506	0.00039438	Tulp1/Hif1a/Ror	14	BP	5.37557562	0.06666667
GO:0042462	eye photoreceptor cell development	6/210	39/18773	4.37E-06	0.00046639	0.00039551	Tulp1/Rpgrip1/(	6	BP	5.35909535	0.02857143
GO:0051654	establishment of mitochondrion localization	5/210	24/18773	5.97E-06	0.00061528	0.00052178	Ubb/Kif1b/Hif1a	5	BP	5.22404373	0.02380952
GO:0005516	calmodulin binding	11/207	183/18634	6.55E-06	0.00141822	0.00122394	Akap12/Myo10/	11	MF	5.1837154	0.0531401
GO:0061351	neural precursor cell proliferation	11/210	182/18773	6.65E-06	0.00066344	0.00056262	Wnt7b/Insm1/C	11	BP	5.17707483	0.05238095
GO:0042670	retinal cone cell differentiation	4/210	12/18773	7.02E-06	0.00067841	0.00057531	Casz1/Gnat2/Ca	4	BP	5.15359737	0.01904762
GO:0008277	regulation of G protein-coupled receptor signaling pathway	9/210	124/18773	1.08E-05	0.00101145	0.00085774	Usp33/Frmpd1/	9	BP	4.96678051	0.04285714
GO:0032103	positive regulation of response to external stimulus	16/210	401/18773	1.22E-05	0.00110945	0.00094085	Lgals9/Cdh13/O	16	BP	4.91365283	0.07619048
GO:0010970	transport along microtubule	10/210	162/18773	1.46E-05	0.00129128	0.00109504	Ubb/Kif1b/Map	10	BP	4.83515036	0.04761905
GO:0001894	tissue homeostasis	12/210	244/18773	2.05E-05	0.00175823	0.00149103	Nxnl2/Tulp1/Pc	12	BP	4.68886081	0.05714286
GO:0099568	cytoplasmic region	11/214	210/18982	2.75E-05	0.00112255	0.00088943	Map2/Gpsm2/D	11	CC	4.56140111	0.05140187
GO:2000177	regulation of neural precursor cell proliferation	8/210	111/18773	3.53E-05	0.00295259	0.00250388	Insm1/Optn/Bd	8	BP	4.45183462	0.03809524
GO:0019897	extrinsic component of plasma membrane	10/214	179/18982	3.70E-05	0.00136161	0.00107885	Cdh13/Rs1/Rtb	10	CC	4.43179585	0.04672897
GO:0051607	defense response to virus	11/210	226/18773	4.99E-05	0.00406132	0.00344411	Ifi44l/Trim30a/F	11	BP	4.30178913	0.05238095
GO:0099111	microtubule-based transport	10/210	188/18773	5.24E-05	0.0041555	0.00352398	Ubb/Kif1b/Map	10	BP	4.2805515	0.04761905
GO:0050839	cell adhesion molecule binding	12/207	271/18634	5.32E-05	0.00768237	0.00662998	Cd9/Cdh13/Anx	12	MF	4.27387151	0.05797101
GO:0009615	response to virus	12/210	270/18773	5.50E-05	0.00425292	0.0036066	Lgals9/Ifi44l/Tri	12	BP	4.25949184	0.05714286
GO:0030705	cytoskeleton-dependent intracellular transport	10/210	191/18773	5.99E-05	0.00451612	0.0038298	Ubb/Kif1b/Map	10	BP	4.22268991	0.04761905
GO:1902904	negative regulation of supramolecular fiber organization	9/210	155/18773	6.34E-05	0.0046701	0.00396038	Capg/Clu/Map2	9	BP	4.19766394	0.04285714
GO:0030659	cytoplasmic vesicle membrane	13/214	323/18982	8.07E-05	0.00232428	0.00184161	Slc10a4/Kif1b/A	13	CC	4.09287582	0.06074766
GO:0042611	MHC protein complex	4/214	21/18982	8.09E-05	0.00232428	0.00184161	H2-DMb2/H2-D	4	CC	4.09222745	0.01869159
GO:0032838	plasma membrane bounded cell projection cytoplasm	9/214	159/18982	8.21E-05	0.00232428	0.00184161	Map2/Dynll1/Pr	9	CC	4.08561565	0.04205607
GO:0006836	neurotransmitter transport	11/210	241/18773	8.89E-05	0.00639104	0.00541979	Slc10a4/Cspg5/	11	BP	4.05119931	0.05238095
GO:0051291	protein heterooligomerization	4/210	22/18773	9.50E-05	0.00667814	0.00566326	Krt1/Mat2a/Ror	4	BP	4.02213111	0.01904762
GO:0045185	maintenance of protein location	7/210	96/18773	0.00010183	0.00693336	0.00587969	Lgals9/Pfn1/Nfk	7	BP	3.99211302	0.03333333
GO:2000179	positive regulation of neural precursor cell proliferation	6/210	67/18773	0.00010315	0.00693336	0.00587969	Insm1/Optn/Hif	6	BP	3.98653792	0.02857143
GO:0035455	response to interferon-alpha	4/210	23/18773	0.00011403	0.00750201	0.00636192	Bst2/Ifitm3/Ifit1	4	BP	3.94296406	0.01904762
GO:0009314	response to radiation	14/210	387/18773	0.00012266	0.00790125	0.00670049	Tulp1/Hif1a/Ror	14	BP	3.91130248	0.06666667
GO:0051646	mitochondrion localization	5/210	45/18773	0.0001418	0.0089476	0.00758782	Ubb/Kif1b/Hif1a	5	BP	3.84833702	0.02380952
GO:0007603	phototransduction, visible light	3/210	10/18773	0.00015627	0.00966357	0.00819499	Rs1/Gnb1/Aipl1	3	BP	3.80613172	0.01428571
GO:0012506	vesicle membrane	13/214	349/18982	0.00017423	0.0045798	0.00362873	Slc10a4/Kif1b/A	13	CC	3.75887323	0.06074766
GO:0042612	MHC class I protein complex	3/214	11/18982	0.00021808	0.00535026	0.00423919	H2-D1/B2m/H2-	3	CC	3.66138203	0.01401869
GO:0150034	distal axon	13/214	374/18982	0.00034011	0.00782262	0.00619813	Clu/Map2/Tulp	13	CC	3.46837582	0.06074766
GO:0005930	axoneme	7/214	117/18982	0.00036484	0.00789768	0.0062576	Prkar2a/Rpgrip1	7	CC	3.43789918	0.03271028
GO:0097014	ciliary plasm	7/214	119/18982	0.00040436	0.00826698	0.00655021	Prkar2a/Rpgrip1	7	CC	3.39322829	0.03271028
GO:0005834	heterotrimeric G-protein complex	4/214	33/18982	0.00049734	0.00915114	0.00725076	Gnb1/Gngt1/Gn	4	CC	3.30334269	0.01869159
GO:1905360	GTPase complex	4/214	33/18982	0.00049734	0.00915114	0.00725076	Gnb1/Gngt1/Gn	4	CC	3.30334269	0.01869159
GO:0042824	MHC class I peptide loading complex	3/214	15/18982	0.00058166	0.00979949	0.00776447	H2-D1/B2m/H2-	3	CC	3.23532753	0.01401869
GO:0044306	neuron projection terminus	9/214	207/18982	0.00058584	0.00979949	0.00776447	Tulp1/Bdnf/Pac	9	CC	3.23222179	0.04205607

Supplementary Table 7, GO pathway analysis with downregulated genes in bipolar cells

ID	Description	GeneRatio	BgRatio	pvalue	p.adjust	qvalue	geneID	Count	GO_type	minus log10	GeneRatio
GO:0003735	structural constituent of ribosome	14/126	153/18634	2.17E-12	7.11E-10	6.76E-10	Rps3/Mrpl9/	14	MF	11.6638891	0.11111111
GO:0022626	cytosolic ribosome	12/126	111/18982	9.72E-12	2.72E-09	2.33E-09	Rps3/Rpl18a	12	CC	11.0123307	0.0952381
GO:0005840	ribosome	15/126	224/18982	2.42E-11	3.03E-09	2.59E-09	Rps3/Mrpl9/	15	CC	10.6154884	0.11904762
GO:0044391	ribosomal subunit	14/126	190/18982	3.24E-11	3.03E-09	2.59E-09	Rps3/Mrpl9/	14	CC	10.4892698	0.11111111
GO:0070181	small ribosomal subunit rRNA binding	4/126	10/18634	4.06E-07	6.65E-05	6.32E-05	Cirbp/Rps3/F	4	MF	6.391939	0.03174603
GO:0022627	cytosolic small ribosomal subunit	6/126	46/18982	5.72E-07	4.00E-05	3.43E-05	Rps3/Rps14/	6	CC	6.24265386	0.04761905
GO:0015934	large ribosomal subunit	8/126	120/18982	1.36E-06	7.62E-05	6.53E-05	Mrpl9/Rpl18	8	CC	5.86646049	0.06349206
GO:0002181	cytoplasmic translation	7/124	88/18773	1.89E-06	0.00389132	0.00377264	Rpl18a/Rpl1	7	BP	5.72461293	0.05645161
GO:0022625	cytosolic large ribosomal subunit	6/126	60/18982	2.83E-06	0.00013224	0.00011334	Rpl18a/Rplp	6	CC	5.54765895	0.04761905
GO:0015935	small ribosomal subunit	6/126	75/18982	1.05E-05	0.00042034	0.00036029	Rps3/Rps14/	6	CC	4.97846236	0.04761905
GO:0042788	polysomal ribosome	4/126	29/18982	3.87E-05	0.00135289	0.00115962	Rpl18a/Rpl1	4	CC	4.41280562	0.03174603
GO:0005844	polysome	5/126	70/18982	0.00010198	0.00317278	0.00271953	Rps3/Rpl18a	5	CC	3.9914755	0.03968254

Supplementary Table 8, GO pathway analysis with downregulated genes in Amacrine cells

ID	Description	GeneRatio	BgRatio	pvalue	p.adjust	qvalue	geneID	Count	GO_type	minus log10	GeneRatio
GO:0043209	myelin sheath	13/175	205/18982	5.89E-08	2.12E-05	1.70E-05	Atp6v1b2/M	13	CC	7.22992979	0.07428571
GO:0022626	cytosolic ribosome	9/175	111/18982	8.84E-07	0.00015905	0.00012743	Rpl37a/Rpl29	9	CC	6.05373931	0.05142857
GO:0003735	structural constituent of ribosome	10/172	153/18634	1.59E-06	0.00060807	0.00054457	Rpl37a/Rpl29	10	MF	5.79810819	0.05813953
GO:0150034	distal axon	15/175	374/18982	2.07E-06	0.00024881	0.00019934	Ptprf/Cplx3/T	15	CC	5.68330675	0.08571429
GO:0044391	ribosomal subunit	10/175	190/18982	1.09E-05	0.00098272	0.00078733	Rpl37a/Rpl29	10	CC	4.96181163	0.05714286
GO:0070382	exocytic vesicle	11/175	254/18982	2.47E-05	0.00177553	0.0014225	Cplx3/Cdk16	11	CC	4.6080042	0.06285714
GO:0005840	ribosome	10/175	224/18982	4.49E-05	0.00269663	0.00216046	Rpl37a/Rpl29	10	CC	4.3473296	0.05714286
GO:0008021	synaptic vesicle	10/175	232/18982	6.04E-05	0.00285688	0.00228885	Cplx3/Cdk16	10	CC	4.21924141	0.05714286
GO:0022627	cytosolic small ribosomal subunit	5/175	46/18982	6.35E-05	0.00285688	0.00228885	Rps29/Rps28	5	CC	4.19731985	0.02857143
GO:0044306	neuron projection terminus	9/175	207/18982	0.0001326	0.00530409	0.00424948	Cplx3/Slc32a	9	CC	3.87744878	0.05142857
GO:0030659	cytoplasmic vesicle membrane	11/175	323/18982	0.0002106	0.00751327	0.0060194	Cplx3/Slc6a9	11	CC	3.67655027	0.06285714
GO:0031252	cell leading edge	12/175	382/18982	0.00022957	0.00751327	0.0060194	Atp6v1b2/Th	12	CC	3.63908071	0.06857143
GO:0030133	transport vesicle	11/175	330/18982	0.00025319	0.00759566	0.00608541	Cplx3/Cdk16	11	CC	3.59655558	0.06285714
GO:0098563	intrinsic component of synaptic vesicle membrane	5/175	64/18982	0.00030894	0.00855533	0.00685427	Cplx3/Slc6a9	5	CC	3.5101226	0.02857143
GO:0043679	axon terminus	8/175	187/18982	0.00034971	0.00899262	0.00720461	Cplx3/Slc32a	8	CC	3.45628834	0.04571429
GO:0012506	vesicle membrane	11/175	349/18982	0.00040709	0.00977015	0.00782755	Cplx3/Slc6a9	11	CC	3.39030996	0.06285714

Supplementary Table 9, GSEA of rod and Müller glia clusters in P23H vs WT retinas

rod upregulated genes-related TFs

NAME	GS<br>follow link to MSigDB	GS D	SIZE	ES	NES	NOM p-val	FDR q-val	FWER p-va	RANK AT MAX	LEADING EDGE
STAT1 HeLa-S3 hg19.grp	STAT1 HeLa-S3 hg19.grp	Detai	31	0.578262	2.281577	0	0.039234	0.033	40	tags=39%, list=8%, signal=39%
TEAD4 SK-N-SH hg19.grp	TEAD4 SK-N-SH hg19.grp	Detai	16	0.673964	2.18223	0.001664	0.072166	0.117	82	tags=69%, list=17%, signal=80%
BATF GM12878 hg19.grp	BATF GM12878 hg19.grp	Detai	15	0.697051	2.159325	0	0.058917	0.142	71	tags=67%, list=15%, signal=76%
TCF12 SK-N-SH hg19.grp	TCF12 SK-N-SH hg19.grp	Detai	18	0.616285	2.081809	0.001634	0.103581	0.3	54	tags=50%, list=11%, signal=54%
IKZF1 GM12878 hg19.grp	IKZF1 GM12878 hg19.grp	Detai	40	0.479374	2.014515	0.005594	0.1647	0.501	87	tags=45%, list=18%, signal=50%
EP300 HeLa-S3 hg19.grp	EP300 HeLa-S3 hg19.grp	Detai	37	0.480367	1.97442	0.004286	0.202274	0.641	75	tags=41%, list=15%, signal=44%
EP300 SK-N-SH hg19.grp	EP300 SK-N-SH hg19.grp	Detai	62	0.410986	1.870132	0.00267	0.458746	0.935	86	tags=37%, list=18%, signal=39%
TEAD4 ECC-1 hg19.grp	TEAD4 ECC-1 hg19.grp	Detai	62	0.408636	1.868547	0.002614	0.404734	0.936	73	tags=35%, list=15%, signal=36%
MEF2A GM12878 hg19.grp	MEF2A GM12878 hg19.grp	Detai	26	0.483251	1.847117	0.015038	0.429853	0.967	134	tags=62%, list=28%, signal=80%
EP300 ECC-1 hg19.grp	EP300 ECC-1 hg19.grp	Detai	23	0.512143	1.831156	0.012461	0.437107	0.976	46	tags=35%, list=9%, signal=37%
NR3C1 A549 hg19.grp	NR3C1 A549 hg19.grp	Detai	43	0.415577	1.777264	0.009859	0.597982	0.998	134	tags=53%, list=28%, signal=67%
SMC3 HepG2 hg19.grp	SMC3 HepG2 hg19.grp	Detai	44	0.413018	1.765342	0.008392	0.599381	0.999	86	tags=41%, list=18%, signal=45%
CTCF GM19240 hg19.grp	CTCF GM19240 hg19.grp	Detai	37	0.433418	1.761241	0.013314	0.57187	1	78	tags=41%, list=16%, signal=45%
STAT3 HeLa-S3 hg19.grp	STAT3 HeLa-S3 hg19.grp	Detai	67	0.371	1.74812	0.006443	0.582685	1	82	tags=34%, list=17%, signal=36%

rod downregulated genes-related TFs

NAME	GS<br>follow link to MSigDB	GS D	SIZE	ES	NES	NOM p-val	FDR q-val	FWER p-va	RANK AT MAX	LEADING EDGE
YY1 HepG2 hg19.grp	YY1 HepG2 hg19.grp	Detai	22	-0.373977	-1.617542	0.040107	1	0.996	220	tags=77%, list=45%, signal=135%
GABPA K562 hg19.grp	GABPA K562 hg19.grp	Detai	35	-0.307584	-1.55343	0.0625	1	1	185	tags=60%, list=38%, signal=90%
RELA GM12891 hg19.grp	RELA GM12891 hg19.grp	Detai	65	-0.259875	-1.54133	0.030172	1	1	200	tags=63%, list=41%, signal=93%
GABPA SK-N-SH hg19.grp	GABPA SK-N-SH hg19.grp	Detai	36	-0.293363	-1.469014	0.056604	1	1	258	tags=78%, list=53%, signal=154%
TCF12 ECC-1 hg19.grp	TCF12 ECC-1 hg19.grp	Detai	27	-0.308242	-1.42344	0.098901	1	1	174	tags=67%, list=36%, signal=98%

Retinal glia (Muller/astrocytes) upregulated genes-related TFs

NAME	GS<br>follow link to MSigDB	GS D	SIZE	ES	NES	NOM p-val	FDR q-val	FWER p-va	RANK AT MAX	LEADING EDGE
JUND H1-hESC hg19.grp	JUND H1-hESC hg19.grp	Detai	24	0.434206	1.731412	0.014002	1	0.999	108	tags=71%, list=36%, signal=102%
JUND HeLa-S3 hg19.grp	JUND HeLa-S3 hg19.grp	Detai	19	0.442398	1.64157	0.027027	1	1	83	tags=58%, list=28%, signal=75%
PRDM1 HeLa-S3 hg19.grp	PRDM1 HeLa-S3 hg19.grp	Detai	23	0.406584	1.624474	0.031432	1	1	82	tags=57%, list=28%, signal=72%
BCL11A GM12878 hg19.grp	BCL11A GM12878 hg19.grp	Detai	16	0.418579	1.524936	0.053284	1	1	92	tags=63%, list=31%, signal=86%
TCF12 myocyte mm9.grp	TCF12 myocyte mm9.grp	Detai	27	0.355985	1.502258	0.068927	1	1	78	tags=44%, list=26%, signal=55%

Retinal glia (Muller/astrocytes) downregulated genes-related TFs

NAME	GS<br>follow link to MSigDB	GS D	SIZE	ES	NES	NOM p-val	FDR q-val	FWER p-va	RANK AT MAX	LEADING EDGE
CTCF testis mm9.grp	CTCF testis mm9.grp	Detai	27	-0.310611	-2.005792	0	0.530678	0.209	171	tags=89%, list=58%, signal=190%
CTCF GM19240 hg19.grp	CTCF GM19240 hg19.grp	Detai	20	-0.311236	-1.661771	0.006024	1	0.833	188	tags=95%, list=63%, signal=241%
MAZ K562 hg19.grp	MAZ K562 hg19.grp	Detai	26	-0.278955	-1.645374	0.008264	1	0.852	126	tags=73%, list=42%, signal=116%
ZNF384 MEL cell line mm9.grp	ZNF384 MEL cell line mm9.grp	Detai	23	-0.264618	-1.58023	0.012658	1	0.92	164	tags=87%, list=55%, signal=179%
NELFE CH12.LX mm9.grp	NELFE CH12.LX mm9.grp	Detai	23	-0.268068	-1.557995	0.049645	1	0.936	208	tags=96%, list=70%, signal=294%
CHD1 H1-hESC hg19.grp	CHD1 H1-hESC hg19.grp	Detai	33	-0.21897	-1.557739	0.018349	1	0.936	187	tags=91%, list=63%, signal=218%
CTCF astrocyte of the spinal cc	CTCF astrocyte of the spinal cor	Detai	29	-0.241039	-1.521745	0.056604	1	0.964	179	tags=86%, list=60%, signal=196%
NFYB K562 hg19.grp	NFYB K562 hg19.grp	Detai	23	-0.259703	-1.516779	0.048276	1	0.969	46	tags=39%, list=15%, signal=43%
POLR2A liver mm9.grp	POLR2A liver mm9.grp	Detai	31	-0.226873	-1.504234	0.025	0.96177	0.973	168	tags=84%, list=57%, signal=173%
CTCF osteoblast hg19.grp	CTCF osteoblast hg19.grp	Detai	24	-0.256033	-1.491946	0.065041	0.922305	0.981	179	tags=88%, list=60%, signal=202%
CTCF skin fibroblast hg19.grp	CTCF skin fibroblast hg19.grp	Detai	17	-0.289571	-1.489897	0.034884	0.847565	0.982	165	tags=88%, list=56%, signal=187%
ZNF384 CH12.LX mm9.grp	ZNF384 CH12.LX mm9.grp	Detai	31	-0.220611	-1.483546	0.065421	0.799175	0.984	161	tags=81%, list=54%, signal=158%
UBTF MEL cell line mm9.grp	UBTF MEL cell line mm9.grp	Detai	35	-0.212534	-1.466708	0.07619	0.801737	0.988	231	tags=97%, list=78%, signal=386%
CTCF fibroblast of villous mese	CTCF fibroblast of villous mesen	Detai	29	-0.219911	-1.450282	0.028846	0.799462	0.991	173	tags=83%, list=58%, signal=179%

Cone upregulated genes-related TFs

NAME	GS<br>follow link to MSigDB	GS D	SIZE	ES	NES	NOM p-val	FDR q-val	FWER p-va	RANK AT MAX	LEADING EDGE
GABPA HL-60 hg19.grp	GABPA HL-60 hg19.grp	Detai	22	0.299145	1.641659	0.007874	1	0.564	103	tags=100%, list=74%, signal=325%
ELK4 HEK293 hg19.grp	ELK4 HEK293 hg19.grp	Detai	18	0.322314	1.540951	0.071429	0.880385	0.715	99	tags=100%, list=71%, signal=303%
GATA3 SH-SY5Y hg19.grp	GATA3 SH-SY5Y hg19.grp	Detai	15	0.330645	1.49429	0.065574	0.726011	0.787	97	tags=100%, list=70%, signal=295%
MAX H1-hESC hg19.grp	MAX H1-hESC hg19.grp	Detai	17	0.308443	1.474285	0.059524	0.600318	0.824	88	tags=94%, list=63%, signal=225%
GABPA GM12878 hg19.grp	GABPA GM12878 hg19.grp	Detai	16	0.300813	1.438642	0.082278	0.559502	0.869	101	tags=100%, list=73%, signal=324%

Cone downregulated genes-related TFs

NAME	GS<br>follow link to MSigDB	GS D	SIZE	ES	NES	NOM p-val	FDR q-val	FWER p-va	RANK AT MAX	LEADING EDGE
MAX A549 hg19.grp	MAX A549 hg19.grp	Detai	18	-0.425527	-1.54213	0.057176	1	1	83	tags=94%, list=60%, signal=204%
POLR2A spleen mm9.grp	POLR2A spleen mm9.grp	Detai	18	-0.425258	-1.541406	0.048555	1	1	75	tags=89%, list=54%, signal=168%
CTCF astrocyte hg19.grp	CTCF astrocyte hg19.grp	Detai	15	-0.446529	-1.538345	0.058683	1	1	73	tags=87%, list=53%, signal=163%
MYC MEL cell line mm9.grp	MYC MEL cell line mm9.grp	Detai	20	-0.418045	-1.535843	0.052692	1	1	71	tags=80%, list=51%, signal=140%
